# Rational Design of New Monoterpene-Containing Azoles and Their Antifungal Activity

**DOI:** 10.3390/antibiotics12050818

**Published:** 2023-04-27

**Authors:** Nikolai S. Li-Zhulanov, Nadezhda P. Zaikova, Suat Sari, Dolunay Gülmez, Suna Sabuncuoğlu, Keriman Ozadali-Sari, Sevtap Arikan-Akdagli, Andrey A. Nefedov, Tatyana V. Rybalova, Konstantin P. Volcho, Nariman F. Salakhutdinov

**Affiliations:** 1 N. N. Vorozhtsov Novosibirsk Institute of Organic Chemistry, Siberian Branch, Russian Academy of Sciences, Lavrentiev Ave., 9, 630090 Novosibirsk, Russia; 2 Department of Pharmaceutical Chemistry, Hacettepe University Faculty of Pharmacy, Sihhiye, Ankara 06100, Turkey; 3 Department of Medical Microbiology, Hacettepe University Faculty of Medicine, Sihhiye, Ankara 06100, Turkey; 4 Department of Pharmaceutical Toxicology, Hacettepe University Faculty of Pharmacy, Sihhiye, Ankara 06100, Turkey

**Keywords:** triazole antifungals, monoterpenoids, hybrids, clinical isolates, CYP51, *Candida* spp.

## Abstract

Azole antifungals, including fluconazole, have long been the first-line antifungal agents in the fight against fungal infections. The emergence of drug-resistant strains and the associated increase in mortality from systemic mycoses has prompted the development of new agents based on azoles. We reported a synthesis of novel monoterpene-containing azoles with high antifungal activity and low cytotoxicity. These hybrids demonstrated broad-spectrum activity against all tested fungal strains, with excellent minimum inhibitory concentration (MIC) values against both fluconazole-susceptible and fluconazole-resistant strains of *Candida* spp. Compounds **10a** and **10c** with cuminyl and pinenyl fragments demonstrated up to 100 times lower MICs than fluconazole against clinical isolates. The results indicated that the monoterpene-containing azoles had much lower MICs against fluconazole-resistant clinical isolates of *Candida parapsilosis* than their phenyl-containing counterpart. In addition, the compounds did not exhibit cytotoxicity at active concentrations in the MTT assay, indicating potential for further development as antifungal agents.

## 1. Introduction

Fungal infections are a major public health concern as about 600 fungi species can cause human disease, and there are no licensed vaccines to prevent them. Fungal diseases have a significant impact on public health, with over one million humans dying every year from them [1]. The problem is compounded by a lack of funding for research, inadequate awareness among public health authorities, reduced global access to antifungals, antifungal resistance, and insufficient alternatives for accurate diagnosis. World Health Organization published WHO Fungal Priority Pathogens List to Guide Research, Development and Public Health Action on 25 October 2022 to underline the need for action [2]. The number of cases of fungal infections is increasing, particularly in immunocompromised patients, and current treatments are expensive and toxic. Additionally, many mycoses require hospitalization, and the most effective drugs for combating them have low availability in regions where fungal infections are prevalent, which is exacerbated by the emergence of species resistant to current therapies [2].

**Figure 1:**
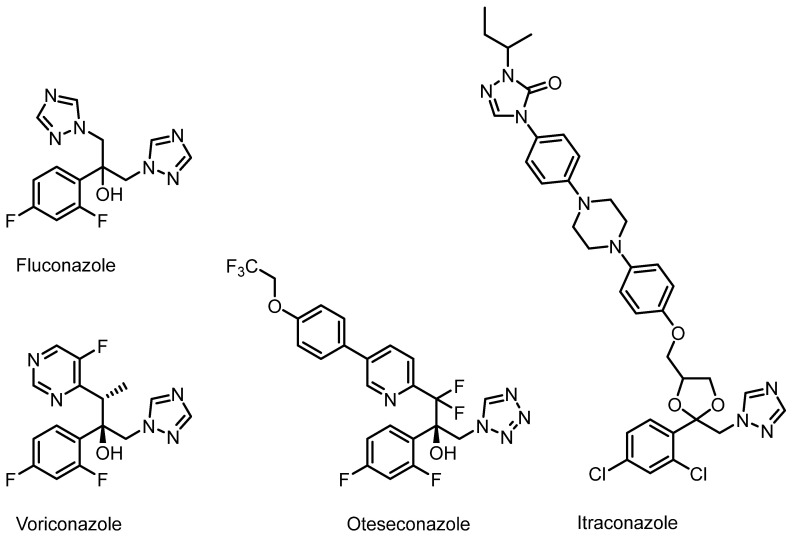
Some azole antifungals.

There are four main families of antifungals: polyenes, azoles, echinocandins, and pyrimidine analogues [3,4,5,6]. However, these antifungals are associated with therapeutic failures and antifungal resistance, making treatment difficult. For example, amphotericin B is highly toxic and expensive [7], and resistance to azoles is increasing [8]. Echinocandins and pyrimidine analogues have also been associated with resistance [3,9]. As a result, researchers look for new drugs and cellular targets. Azoles have long been first-line antifungals in fighting mycoses. The emergence of drug-resistant strains and the associated increase in mortality of systemic mycoses led to the development of new-generation, broad-spectrum triazole and tetrazole antifungals. Fluconazole, itraconazole, voriconazole, and oteseconazole are among the currently used azole antifungals with systemic availability and broad-spectrum efficacy (Figure 1) [10,11].

The COVID-19 pandemic caused by SARS-CoV-2 has made the relationship between fungal diseases and public health even more complex. Fungal diseases were mostly associated with COVID-19 in individuals with weakened immune systems in intensive care units [12].

The process of discovering and developing a drug candidate for fungal diseases is time-consuming and costly, and current methods such as combinatorial chemistry and virtual screening have partly been successful [13]. Hybrid drug discovery, which combines two or more chemical scaffolds that act on different targets to create more specific and powerful drugs, is gaining momentum as a potential solution for developing new antifungal drugs that are affordable and can avoid the emergence of resistant strains. Molecular hybrids are a promising tool in medicinal chemistry efforts for drug discovery [14].

Natural products are a dominant source of biologically active structures, and the hybridization of these structures with fluconazole can lead to potent drugs [15]. Zhang et al. [16] reported the design and synthesis of novel series of carbazole-triazole conjugates. Elias et al. [17], in an effort to synthesize new azole hybrids with a coumarin scaffold, showed that hybrid antifungals could penetrate the endoplasmic reticulum of fungal cells where access to azole antifungal target, lanosterol 14-alpha demethylase (CYP51), is available. This approach can potentially enhance the efficacy of azole antifungal drugs.

**Figure 2:**
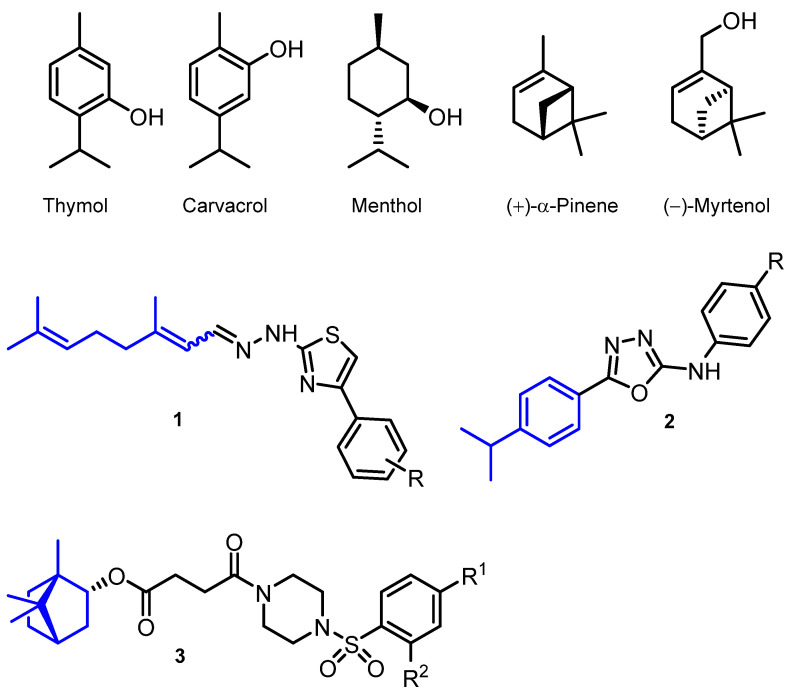
Monoterpenes and monoterpene-based compounds with antifungal activity.

Although the use of natural compounds as a basis for the development of new drugs remains one of the most important areas of modern organic chemistry [18], the role of monoterpenes in this direction is not leading, which is largely due to the numerous synthetic difficulties that researchers working in this field face. At the same time, monoterpenes and monoterpenoids demonstrated promising fungicidal properties so far [19]. In particular, monoterpenes were reported to have antifungal activity both alone and in combination with antifungal agents [20,21,22,23]. For example, Ahmad A. et al. showed a synergistic effect against *Candida* spp. by combined use of fluconazole and monoterpene phenols, thymol, and carvacrol (Figure 2) [20]. In addition, studies were recently conducted on the antifungal activity of a wide range of monoterpenes of various structures (e.g., (+)-α-pinene, menthol, (−)-myrtenol, etc.), some of which showed significant activity against *Candida* spp. with minimal inhibition concentration (MIC) values ranging between 23–51 µg/mL [23,24].

In addition, several compounds with antifungal properties were synthesized based on monoterpenes (Figure 2). Citral-thiazolyl hydrazine derivatives **1** showed potent antifungal activity, for example, against *Rhizoctonia solani*, with EC_50_ values of 0.640 µg/mL [25]. Plant cuminaldehyde-derived oxadiazole **2** derivatives inhibited *Candida albicans* and *Candida auris* (MICs 0.5–12.0 µg/mL) and showed potent binding with the *C. albicans* CYP51 (CaCYP51) [26]. (−)-Borneol derivatives **3** containing aryl-sulfonamide scaffold displayed potential fungicidal activities against *Botrytis cinerea*, *Curvularia lunata*, and *Alternaria altanata* (*C. lunata* IC_50_ = 22.9 µg/mL) [27]. Isopulegol-based *O*-benzyl derivatives containing imidazole and triazole substituents were found to exhibit marked growth inhibition against *Candida albicans* and *Candida krusei* [28]. Therefore, we assumed that combining the azole core with monoterpenes could yield highly promising new antifungal compounds.

**Figure 3:**
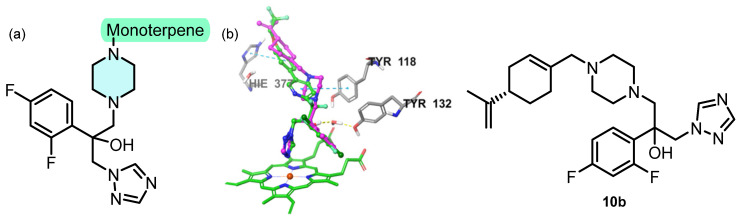
(**a**) General structures of the title azoles. (**b**) The co-crystalized conformer of oteseconazole (green sticks) and the predicted binding mode of proposed azole **10b** (magenta sticks) aligned in CaCYP51 (PDB ID: 5TZ1 [31]) active site. Heme cofactor (green sticks) and heme iron (red sphere) are displayed along with the amino acid residues in polar contacts with the ligands.

Azole antifungals are known to act by inhibiting fungal CYP51, thus inhibiting the biosynthesis of ergosterol, a vital component of fungal membranes. Azoles incorporate three key pharmacophores: an azole ring, a substituted phenyl linked to the azole ring by an ethylene bridge, and a tail group attached to the ethylene linker [29]. CYP51 substrate binding domain, like other CYPs, includes a heme co-factor with an oxidizing iron that makes six coordination bonds [30]: four equatorial bonds with the protoporphyrin, one axial bond with a cysteine side chain, and another axial bond with oxygen, which is used to oxidize the substrate close by. Azoles compete with the natural ligand, lanosterol, and tightly bind the CYP51 active site. The azole ring makes axial coordination with the heme iron replacing the oxygen, while the substituted phenyl contacts the residues close to the heme and the tail occupies the narrow tunnel opening to the catalytic site [31,32]. Preliminary molecular modeling for the proposed monoterpene-containing azole **10b** (Figure 3b) was performed, and it was found that this compound showed excellent binding to CaCYP51 with all the pharmacophores in their places in agreement with the known azoles (Figure 3b), showing that monoterpene substituted azoles could be a promising series. To test this hypothesis, we designed novel monoterpene-azole hybrids that combine the monoterpene scaffold and azole core through a piperazine linker (Figure 3a) and evaluate their antifungal activity.

## 2. Results and Discussion

### 2.1. Chemistry

**Scheme 1:**
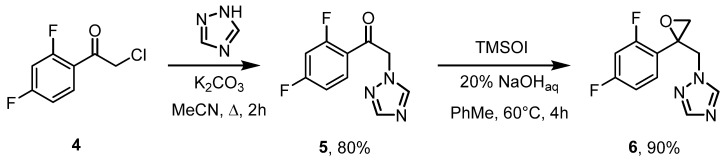
Synthesis of oxirane **6**.

Triazole-containing oxirane **6** was prepared in two steps from the commercially available fluorinated compound **4** according to the procedure [33] with minor modifications (Scheme 1). Alkylation of 1,2,4-triazole by chloroketone **4** gave ketone **5** with an 80% yield. The carbonyl group of compound **5** was then converted to epoxide by using trimethylsulfoxonium iodide (TMSOI) in the presence of a strong base, which resulted in oxirane **6** with a 90% yield.

**Scheme 2:**
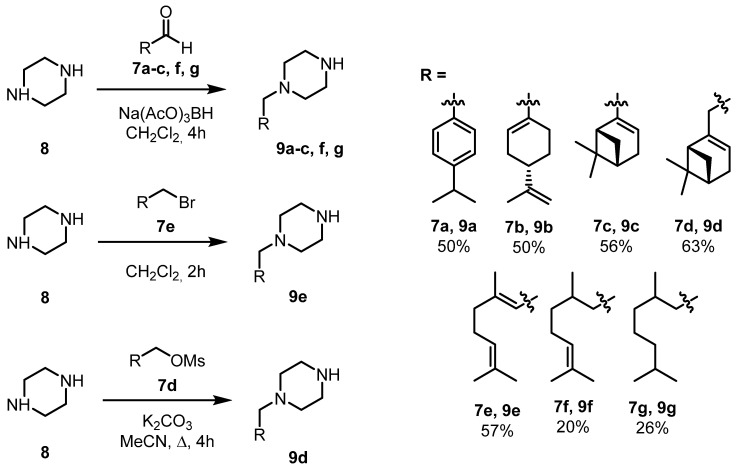
Synthesis of monoterpene-piperazine building blocks **9a**–**g**.

Monoterpene-piperazine building blocks **9a**–**g** were synthesized from the corresponding monoterpene derivatives, namely aldehydes **7a**–**c, f, g**, bromide **7e** and mesylate **7d** (Scheme 2). The reactions of monoterpene aldehydes **7a**–**c, f, g** with an excess of piperazine **8** generated derivatives **9a**–**c, f, g** with moderate yields. 3,7-Dimethyloctanal **7g** was synthesized from 3,7-dimethyloctan-1-ol by the procedure described in [34]. The nucleophilic substitution reactions of piperazine **8** with geranyl bromide **7e** resulted in product **9e** with 57% yield. For the synthesis of **9d**, (−)-nopol was converted to mesylate **7d** according to [35] and then reacted with piperazine. All synthesized monoterpene-piperazines were purified by column chromatography.

**Scheme 3:**
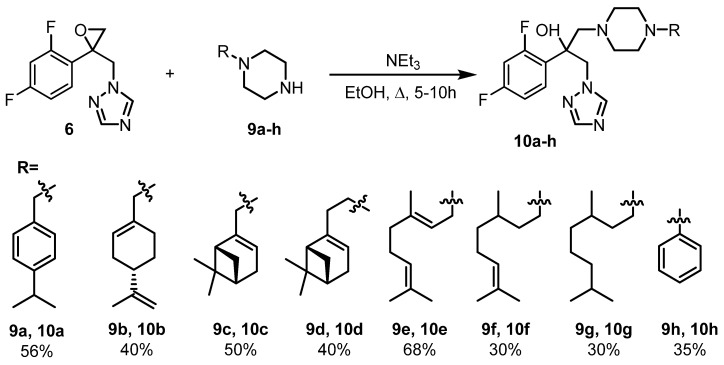
Synthesis of target azoles **10a**–**h**.

Next, two building blocks **6** and **9a**–**g** were reacted in boiling ethanol under mild basic conditions to afford monoterpene-containing azoles **10a**–**g** with satisfactory yields of 30–68% (Scheme 3). Similarly, phenyl-containing azole **10h** was synthesized from commercially available 1-phenylpiperazine **9h**. The low yield of **10f**–**h** is associated with the incomplete conversion of the reagents. However, when trying to carry out the reaction for a longer time, undesirable side reactions were observed. The target hybrids were purified by column chromatography and isolated as an equimolar mixture of enantiomers (**10a**, **10e**, and **10h**) or diastereomers (**10b**–**d, f, g**) and were used in the biological tests as is.

**Figure 4:**
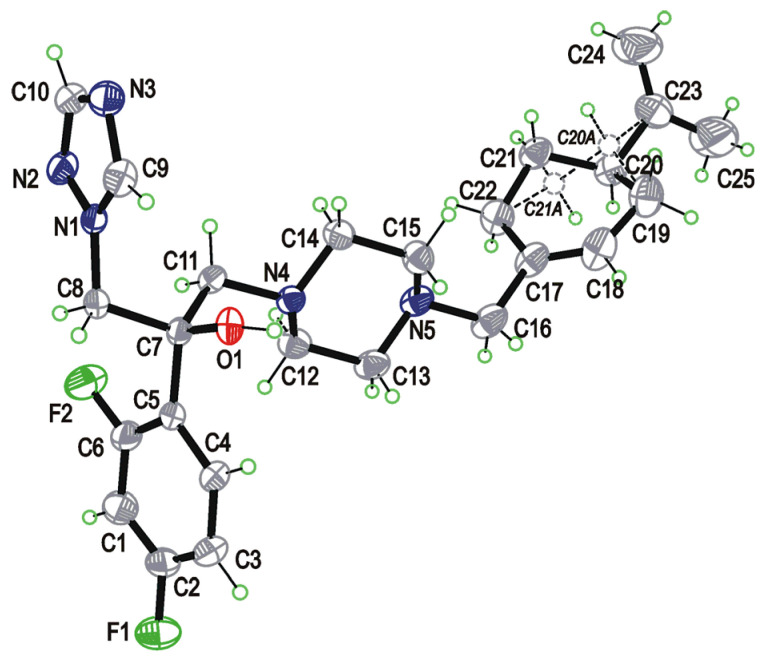
The molecular structure of 2-(2,4-Difluorophenyl)-1-(4-(((*S*)-4-(prop-1-en-2-yl)cyclohex-1-en-1-yl)methyl)piperazin-1-yl)-3-(1*H*-1,2,4-triazol-1-yl)propan-2-ol **10b** with atom-labeling scheme (Displacement ellipsoids are drawn at 30% probability level. The disordered part of molecule (C20A, C21A, belonging to epimer on C20) is shown in dotted lines).

For X-ray structural analysis, single crystals were grown from a solution of compound **10b** in MeOH (Figure 4). Crystals of **10b** shows triclinic space group P-1, *a* 6.5543(4), *b* 10.5843(8), *c* 18.895(1) Å, *α* 104.242(3), *β* 93.514(3), *γ* 92.059(3)°, *V* 1266.4(2) Å^3^, Z 2, C_25_H_33_N_5_OF_2_, Dc 1.200 g/cm^3^, μ 0.086 mm^−1^, F(000) 488, crystal size 0.90 × 0.33 × 0.05 mm^3^, independent reflections 5827, R 0.0793, wR2 0.1757, S 1.052 for all reflections (R 0.0606, wR2 0.1629 for 4422 I > 2σ), largest difference peak and hole 0.231 and −0.278 e.Å^−3^.

Whereas crystals of **10b** belong to centrosymmetric space group P-1, they are a racemic mixture of enantiomers. Each of the enantiomers, in turn, has an epimeric pair on center C20 in an approximate ratio of 1:1, which is proved by the disordering of atoms C20 and C21 in two positions (C20A and C21A, accordingly). The formation of four optical isomers mixture can be due to the enantiomeric purity of the starting L-(−)-perillaldehyde, which is 95%.

A total of seven monoterpene-containing azoles and one phenyl-containing azole were prepared and characterized by ^1^H-NMR, ^13^C-NMR and HR-MS. NMR spectra were recorded for a mixture (1:1) of enantiomers (**10a**, **10e**, and **10h**) or diastereomers (**10b**–**d, f, g**). Our attempts to separate diastereomers by chromatography were unsuccessful (Copies of ^1^H and ^13^C NMR Spectra can be found in the Supplementary Materials).

### 2.2. Biology

We evaluate the antifungal activity of these compounds against a variety of *C. albicans* strains in vitro by determining their minimum inhibitory concentration (MIC) values. The synthesized azoles were first tested against seven American Type Culture Collection (ATCC) fungal strains of *Candida*, and all of them demonstrated excellent activity with MIC values much better than those of fluconazole (FCZ) (Table 1). MIC values of novel azoles were at least 22-fold lower than fluconazole MICs in >90% of the tests with both reference methods. Azoles with fragments of cuminaldehyde **10a**, myrtenal **10c**, and nopol **10d** were the most active with the lowest MICs. It can be noted that azoles containing a cyclic fragment **10a**–**d, h** are more active than linear ones **10e**–**g**.

Further, most promising monoterpene-containing azoles **10a**, **10c**, and, for comparison, phenyl-containing azole **10h** were tested against fluconazole-susceptible, fluconazole-susceptible-at-increased-exposure and fluconazole-resistant clinical isolates including *Candida parapsilosis* and *Candida glabrata*. It is worth noting the difference in activity against clinical isolates for monoterpene-containing azoles **10a** and phenyl-containing azole **10h**. All three compounds had quite similar MICs against standard strains of *C. parapsilosis*; however, monoterpene-containing azoles had much lower MICs against the fluconazole-resistant clinical isolate of *C. parapsilosis* than their aromatic counterpart (Table 2).

**Figure 5:**
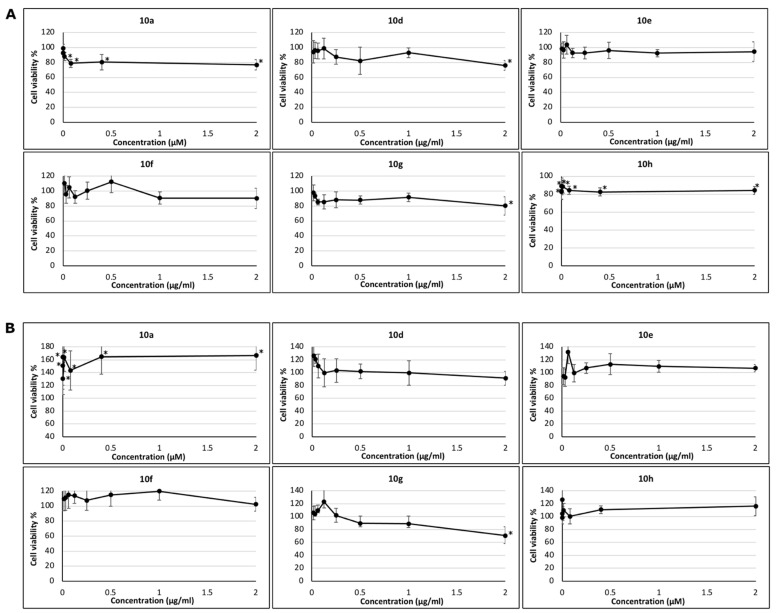
Cell viability % versus compound concertation with respect to the control after 24 (**A**) and 48 h (**B**) upon treatment of the cells with increasing concentrations of the title azoles (* *p* ≤ 0.005).

Compounds **10a** and **10d**–**h** were evaluated for possible cytotoxic effects on murine fibroblasts at varying concentrations, including their MIC values, using the MTT assay. The method simply demonstrates the viability percentage of the cells treated with the compounds compared to the control (non-treated cells). The cells were observed to be most robust after 24 and 48 h of azole treatment, especially at concentrations close to their MIC values. There was negligible viability decline at high concentrations of **10a**, **10d**, and **10g** at 24 h (Figure 5). (The mean and standard deviation values can be found in the Supplementary Materials File).

Briefly, these monoterpene-containing azoles displayed excellent activities against *Candida* spp., including standard strains and clinical isolates, indicating that these compounds are worthwhile to be explored as potential broad-spectrum antifungal agents.

### 2.3. Molecular Modeling Study

#### 2.3.1. Prediction of Druglikeness

**Figure 6:**
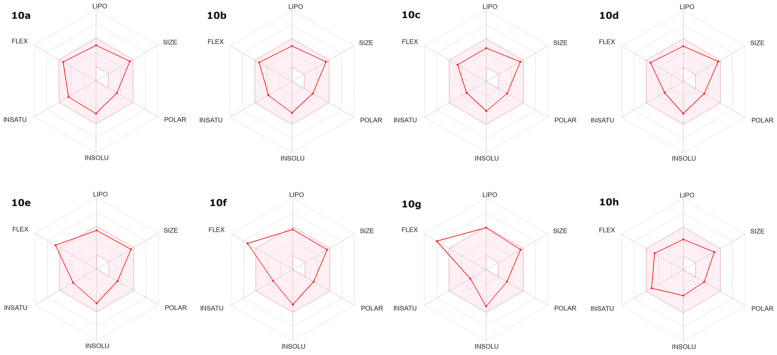
Druglikeness radar graph created for the title azoles by SwissADME [39]. The graph shows a pink hexagon representing druglike chemical space in terms of ideal values for the six descriptors (LogP: between −0.7 and +5.0, MW: between 150 and 500 g/mol, TPSA: between 20 and 130 Å^2^, log S: ≤6, fraction of sp^3^-hybridized carbons: not less than 0.25, and the number of rotatable bonds: ≤9) with each descriptor representing a corner.

Druglikeness of the title azoles was evaluated using six common descriptors suggested to define druglike chemical space, namely LogP for lipophilicity, molecular weight (MW) for size, total polar surface area (TPSA) for polarity, log S for aqueous solubility, the fraction of sp^3^-hybridized carbons over the total carbon count for saturation, and the number of rotatable bonds for flexibility, as calculated by SwissADME [37,38]. The results suggested that **10a**–**d** and **10h** had excellent balance for size, polarity, lipophilicity, aqueous solubility, flexibility, and saturation (Figure 6). Compounds **10e**–**g** were predicted out of druglike chemical space in terms of flexibility since these compounds have too many rotatable bonds due to the monoterpenes used for the tail section, which could be an important factor leading to lower MIC values for these compounds compared to the other title azoles since druglikeness parameters are considered to be associated with the drug concertation available at the target cite for the ultimate pharmacodynamic effects. In addition, the title azoles were free of non-specific reactive fragments, highly available through oral intake, and permeable through skin, as the predictions suggested (see Supporting Information for details).

#### 2.3.2. Predicted Binding of **10a**–**h** to CaCYP51

Prior to the docking of the title azoles, a brief validation study was performed to test the predictive ability of the docking method by redocking the co-crystallized ligand in the CaCYP51 structure, oteseconazole. The predicted binding mode of oteseconazole (docking score −9.5 kcal/mol) was very close to its co-crystallized conformer with an RMSD (root-mean-square deviation) value of 1.24 Å, showing the reliability of the method.

**Figure 7:**
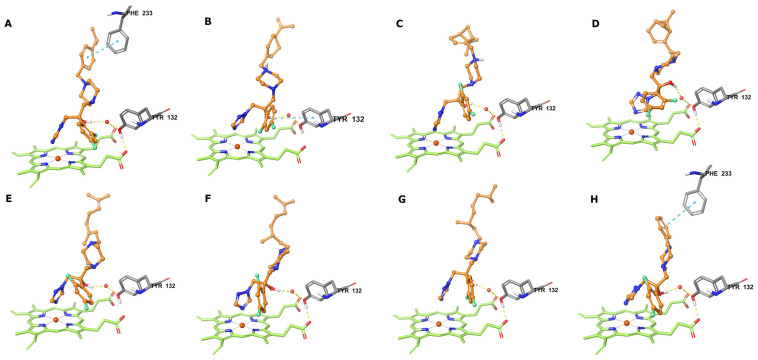
Predicted binding modes of **10a**–**h** ((**A**–**H)**, respectively) in CaCYP51 substrate binding cavity. Ligands are represented as orange sticks, amino acid residues as gray sticks, heme iron as red spheres, and H bond interactions as dashed lines.

**Figure 8:**
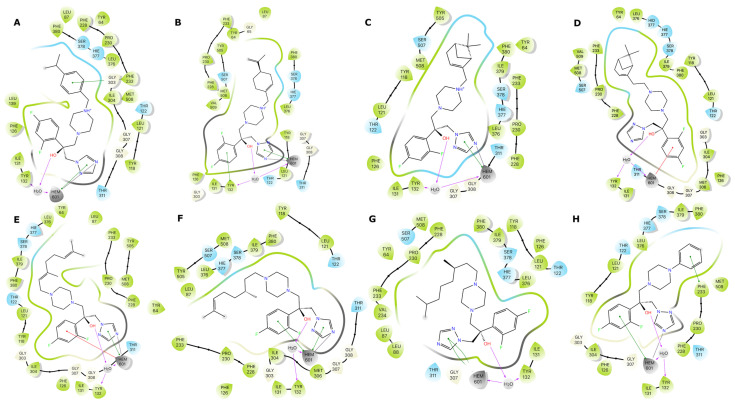
Two-dimensional interactions diagrams for the predicted binding modes of **10a**–**h** with CaCYP51 ((**A**–**H)**, respectively). All the residues within contact distance are shown, electrostatic interactions are represented as color lines/arrows, and vdW contacts are indicated as color bands around each ligand.

The title compounds were predicted to show high affinity to the CaCYP51 substrate binding cavity compared to fluconazole, with docking scores ranging between −7.5 and −9.4 kcal/mol (Table 3). There was a consensus among the predicted binding modes of the title azoles (Figure 7). The triazole ring engaged in axial coordination with the heme iron via N^4^. The difluorophenyl ring fit in the pocket between the heme and a group of nonpolar residues, including Phe 126, Ile 131, Tyr 132, and Gly 303 (Figure 8). It was observed to stack with the heme in the case of **10d**–**f** and **10h**. The tail effectively occupied the active site gorge mainly through hydrophobic interactions. Those including an aromatic ring in the tail were in pi-pi stacks with Phe 233, one of the key residues in the active site [31,40]. Of note was the water-mediated H bond with Tyr 132 through the hydroxyl of all the title azoles (Figure 7 and Figure 8). Mutation studies in *Saccharomyces cerevisiae* analogous to Y132F/H suggest the water-mediated H bond with the tertiary alcohol of certain azoles, including fluconazole, oteseconazole, and voriconazole, is critical for the potency of these agents [41].

## 3. Materials and Methods

### 3.1. Chemistry

General Information: Cuminaldehyde > 98% (SAFC, Germany), (1*R*)-(−)-myrtenal > 97% (*ee* 95%), (1*R*)-(−)-nopol 98% (*ee* 95%), L-(−)-perillaldehyde 90% (*ee* 95%), geranyl bromide 95%, 3,7-dimethyloctan-1-ol > 98% (Sigma-Aldrich (St. Louis, MO, USA)), citronellal 85–90% (Fluka, Switzerland), other reagents and solvents were purchased from commercial suppliers (Sigma-Aldrich (St. Louis, MO, USA), Acros (Waltham, MA, USA)) and used as received. Column chromatography (CC): silica gel (SiO_2_; 60–200 μ; Macherey-Nagel (Dueren, Germany)); hexane/EtOAc 100:0 → 0:100 and MeOH/CH_2_Cl_2_ 100:0 → 50:50. GC-MS: Agilent 7890A gas chromatograph (Santa Clara, CA, USA) equipped with a quadrupole mass spectrometer Agilent 5975C as a detector; quartz column HP-5MS (copolymer 5% diphenyl and 95% dimethylsiloxane) of length 30 m, internal diameter 0.25 mm, and stationary phase film thickness 0.25 µm. Optical rotation: polAAr 3005 spectrometer (Optical Activity LTD, Huntingdon, UK). ^1^H and ^13^C NMR: Bruker DRX-500 apparatus at 500.13 MHz (^1^H) and 125.76 MHz (^13^C), Bruker Avance-III 600 apparatus (Billerica, MA, USA) at 600.30 MHz (^1^H) and 150.95 MHz (^13^C) and Bruker Avance 400 (Bruker Corporation, Karlsruhe, Germany) apparatus 400.13 MHz (^1^H) and 100.61 MHz (^13^C) *J* in Hz; structure determinations by analyzing the ^1^H NMR spectra, including ^1^H–^1^H 2D homonuclear correlation, *J*-modulated ^13^C NMR spectra (JMOD), and ^13^C–^1^H 2D heteronuclear correlation with one-bond and long-range spin-spin coupling constants (C-H COSY, ^1^*J*(C,H) = 135 Hz; HSQC, ^1^*J*(C,H) = 145 Hz; HMBC, ^2,3^*J*(C,H) = 7 Hz). HR-MS: DFS Thermo Scientific spectrometer (Waltham, MA, USA) in a full scan mode (15–500 m/z, 70 eV electron impact ionization, direct sample administration); Bruker Daltonics GmbH micrOTOF-Q spectrometer (Bremen, Germany), electrospray ionization, positive ion mode. Agilent 6890N gas chromatograph (Santa Clara, CA, USA) equipped with a quadrupole mass spectrometer Agilent 5973 N as a detector; chiral column Cyclosil-B (30 m × 250 µm × 0.25 µm). X-ray structural analysis of a single crystal was performed on a Bruker KAPPA APEX II diffractometer (Madison, WI, USA) at 296(2) K. Melting points were determined using a Mettler Toledo FP900 Thermosystem (Greifensee, Switzerland).

Spectral and analytical investigations were carried out at the Multi-Access Chemical Research Center of the SB RAS. All the target compounds reported in this paper have a purity of at least 95%.

#### 3.1.1. Synthesis of Oxirane **6**

A mixture of **4** (1.0 g, 5.3 mmol), 1,2,4-triazole (0.73 g, 10.6 mmol), K_2_CO_3_ (0.73 g, 5.3 mmol) in MeCN (20 mL) was refluxed for 2 h. After the reaction was completed, the reaction mixture was washed with H_2_O (2 × 20 mL) and brine (20 mL) and dried over anhydrous Na_2_SO_4_. The desiccant was filtered. The filtrate was then concentrated under reduced pressure, and the product was recrystallized from a mixture of hexane/EtOAc 1:1 to yield compound **5** as a white solid. The yield was 0.95 g (80%). Then, to a solution of **5** (0.50 g, 2.24 mmol) in toluene (5 mL) was added trimethylsulfoxonium iodide (0.59 g, 2.68 mmol) followed by the addition of 20% sodium hydroxide solution (1 mL). The reaction mixture was then heated at 60 °C for 4 h. After the reaction was over, it was diluted with EtOAc (10 mL), washed with H_2_O (2 × 10 mL) and brine (10 mL), dried over Na_2_SO_4_, and filtered. The filtrate was concentrated under reduced pressure to give **6** as a light brown oil. The yield was 0.48 g (90%). Experimental data of **5** and **6** are consistent with the previous results [33].

#### 3.1.2. Synthesis of 3,7-Dimethyloctanal **7g**

A solution of 3,7-dimethyloctan-1-ol (1.0 g, 6.3 mmol) in 15 mL CH_2_Cl_2_ was added to a stirring suspension of pyridinium chlorochromate (2.0 g, 9.5 mmol) in 65 mL of CH_2_Cl_2_. Stirring was continued for 2 h. The mixture was diluted with Et_2_O (20 mL) and filtered through a silica gel pad. The residue was washed with Et_2_O (20 mL), and the filtrate was evaporated. The residue was purified by column chromatography on silica gel using CH_2_Cl_2_ as the eluent to give aldehyde **7g** (0.9 g, 90%). Experimental data of **7g** are consistent with the previous results [34].

#### 3.1.3. Synthesis of (−)-Nopol Mesylate **7d**

The (−)-nopol (0.50 g, 3.0 mmol) and Et_3_N (0.43 mL, 3.1 mmol) were dissolved in 10 mL of CH_2_Cl_2_. A solution of methanesulfonyl chloride (0.24 mL, 3.1 mmol) in 10 mL of CH_2_Cl_2_ was added at 0 °C within 30 min and stirred. The mixture was further stirred at room temperature for 4 h. The reaction was quenched by a saturated solution of NaHCO_3_, and the aqueous phase was extracted with CH_2_Cl_2_ (3 × 20 mL). The collected organic phases were washed with brine (2 × 20 mL) and dried over Na_2_SO_4_ and filtered. The filtrate was concentrated under reduced pressure obtaining 0.78 g (3.2 mmol) of **7d**. with a yield of 98%. Experimental data of **7d** are consistent with the previous results [35].

#### 3.1.4. Synthesis of Monoterpene-Piperazines **9a**–**c**, **f**, **g**

General procedure: Corresponding aldehyde **7a**–**c, f, g** (1 eq) and piperazine **8** (5 eq) were dissolved in dry CH_2_Cl_2_ (80 mL). The reaction mixture was stirred at r.t. for 30 min, and then Na(AcO)_3_BH (1.5 eq) was added. After 4 h, the reaction was quenched with a saturate solution of NaHCO_3_ (50 mL). The product was extracted with CH_2_Cl_2_ (3 × 20 mL). The extracts were washed with brine, dried with Na_2_SO_4_, and evaporated. The products were isolated by column chromatography on silica gel, eluent MeOH in CH_2_Cl_2_ from 0% to 50%. ^1^H and ^13^C NMR data for compounds **9a** and **9f** correspond to those published earlier [42,43].

*(S)-1-((4-(Prop-1-en-2-yl)cyclohex-1-en-1-yl)methyl)piperazine* **9b**. Yield 40%. Yellow oil. ${\left[\alpha \right]}_{D}^{27.3}$ = −60.5 (*c* 0.40, CHCl_3_). HRMS: 220.1935 [M]^+^; calcd. 220.1934 (C_14_H_24_N_2_). ^1^H-NMR (CDCl_3_, δ ppm, *J*, Hz): 5.44 (m, 1H, H-6), 4.56 (m, 2H, H-8), 4.33–4.27 (m, 1H, NH), 2.85–2.75 (m, 4H, H-12, H-13), 2.74–2.63 (m, 2H, H-10), 2.34–2.12 (m, 4H, H-11, H-14), 2.03–1.73 (m, 5H, H-2, H-5, H-4), 1.75 (dm, *J* = 12.0, 1H, H-3), 1.60 (s, 3H, H-9), 1.37–1.24 (m, 1H, H-3′). ^13^C-NMR (CDCl_3_, δC): 149.6 (C-1), 134.1 (C-7), 124.2 (C-6), 108.3 (C-8), 65.7, (C-10), 53.2 (C-11, C-14), 45.3 (C-12, C-13), 42.0 (C-4), 30.4 (C-5), 27.5 (C-2), 27.3 (C-3), 20.6 (C-9).

*1-(((1R,5S)-6,6-Dimethylbicyclo[3.1.1]hept-2-en-2-yl)methyl)piperazine* **9c**. Yield 50%. Light yellow solid. Melting point: 58.2 °C with decomposition. ${\left[\alpha \right]}_{D}^{27.3}$ = −5.9 (*c* 0.57, CHCl_3_). HRMS: 220.1937 [M]^+^; calcd. 220.1934 (C_14_H_24_N_2_). ^1^H-NMR (CDCl_3_, δ ppm, *J*, Hz): 5.33–5.29 (m, 1H, H-2), 3.06 (br.s, 1H, NH), 2.87–2.79 (m, 5H, H-11, H-14, H-10), 2.73–2.68 (m, 1H, H-10′), 2.39– 2.26 (m, 5H, H-7, H-12, H-13), 2.26–2.11 (m, 3H, H-6, H-3), 2.06–2.00 (m, 1H, H-4), 1.22 (s, 3H, H-8), 1.06 (d, *J* = 8.4, 1H, H-7′), 0.77 (s, 3H, H-9). ^13^C-NMR (CDCl_3_, δC): 145.0, (C-1), 119.8 (C-2), 64.8 (C-10), 54.2 (C-11, C-14), 45.6 (C-12, C-13), 44.2 (C-6), 40.7 (C-4), 37.8 (C-5), 31.6 (C-7), 31.2 (C-3), 26.1 (C-8), 20.9 (C-9).

*1-(3,7-Dimethyloctyl)piperazine* **9g**. Yield 30%. Colorless oil. HRMS: 226.2401 [M]^+^; calcd. 226.2404 (C_14_H_30_N_2_). ^1^H-NMR (CDCl_3_, δ ppm, *J*, Hz): 2.87–2.80 (m, 4H, H-12, H-13), 2.40–2.30 (m, 4H, H-11, H-14), 2.30–2.20 (m, 2H, H-1), 1.50–1.31 (m, 3H, H-3, H-4, H-7), 1.27–1.01 (m, 7H, H-4′, H-2, H-5, H-6), 0.75–0.83 (m, 9H, H-8, H-9, H-10). ^13^C-NMR (CDCl_3_, δC): 57.2 (C-1), 54.3 (C-11, C-14), 45.7 (C-12, C-13), 39.0 (C-6), 37.2 (C-5), 33.5 (C-4), 31.2 (C-3), 27.7 (C-7), 24.4 (C-2), 22.4 (C-8), 22.3 (C-9), 19.6 (C-10).

#### 3.1.5. Synthesis of Monoterpene-Piperazines **9d**

A mixture of **7d** (0.78 g, 3.2 mmol), piperazine (1.35 g, 15.9 mmol), K_2_CO_3_ (0.44 g, 3.2 mmol) in MeCN (30 mL) was refluxed for 4 h. After the reaction was completed, the reaction mixture was washed with H_2_O (2 × 20 mL), brine (20 mL), and dried over anhydrous Na_2_SO_4_. The desiccant was filtered, and the filtrate was concentrated under reduced pressure. The product was isolated by column chromatography on silica gel, eluent MeOH in CH_2_Cl_2_ from 0% to 50%. Yield 0.47 g (63%).

*1-(2-((1R,5S)-6,6-Dimethylbicyclo[3.1.1]hept-2-en-2-yl)ethyl)piperazine* **9d**. Yield 40%. Colorless oil. ${\left[\alpha \right]}_{D}^{22.0}$ = −25.7 (*c* 0.42, CHCl_3_). HRMS: 234.2093 [M]^+^; calcd. 234.2091 (C_15_H_26_N_2_). ^1^H-NMR (CDCl_3_, δ ppm, *J*, Hz): (mixture of rotamers) 5.16 (s, 1H, H-2), 2.88–2.78 (m, 4H, H-12, H-15), 2.42–2.04 (m, 10H, H-13, H-14, H-10, H-7, H-3), 2.04–1.93 (m, 2H, H-6, H-4), 1.69 (br.s, 1H, NH), 1.21 and 1.97 (s, 3H, H-8), 1.10–1.05 (m, 1H, H-7′), 0.77 and 0.76 (s, 3H, H-9). ^13^C-NMR (CDCl_3_, δC): 146.2 (C-1), 116.8 (C-2), 57.2 (C-11), 54.4 (C-12, C-15), 46.0 (C-13, C-14), 45.8 (C-6), 40.6 (C-4), 37.8 (C-5), 34.0 (C-10), 31.5 (C-7), 31.1 (C-3), 26.1 (C-8), 21.1 (C-9).

#### 3.1.6. Synthesis of Monoterpene-Piperazines **9e**

Geranyl bromide **7e** (0.31 g, 1.4 mmol) and piperazine **8** (0.61 g, 7.1 mmol) were dissolved in dry CH_2_Cl_2_ (10 mL). The reaction mixture was stirred at r.t. for 2 h. Then, the reaction was quenched with a saturated solution of NaHCO_3_ (20 mL). The product was extracted with CH_2_Cl_2_. The extracts were washed with brine (3 × 20 mL), dried with Na_2_SO_4_, and evaporated. The product was isolated by column chromatography on silica gel, eluent MeOH in CH_2_Cl_2_ from 0% to 50%, with a yield of 0.47 g (68%). ^1^H and ^13^C spectra are consistent with the previous results [44].

#### 3.1.7. Synthesis of Monoterpene-Containing Azoles **10a**–**h**

General procedure: To a solution of oxirane **6** (1 eq) in 15 mL of ethanol corresponding monoterpene-piperazine **9a**–**g** or 1-phenylpiperazine **9h** (1.2 eq) and NEt_3_ (2.5 eq) were added. The reaction mixture was refluxed until the TLC analysis (1:1 hexane/EtOAc) demonstrated the total consumption of oxirane **6**. Then, the reaction mixture was washed with brine (3 × 20 mL) and dried over anhydrous Na_2_SO_4_. The desiccant was filtered, the filtrate was concentrated under reduced pressure and the product was purified by column chromatography on silica gel, eluent EtOAc in hexane from 0% to 100%. NMR spectra were recorded for a mixture (1:1) of enantiomers (**10a**, **10e**, and **10h**) or diastereomers (**10b**–**d, f, g**), we cannot distinguish signals from diastereomers in a mixture; therefore, the designations (H-1, H-1ds) and (C-1, C-1ds) are used for different diastereomer signals.

*2-(2,4-Difluorophenyl)-1-(4-(4-isopropylbenzyl)piperazin-1-yl)-3-(1H-1,2,4-triazol-1-yl)propan-2-ol* **10a**. Yield 56%. Light yellow solid. Melting point: 112.4–113.9 °C. HRMS: 456.257 [M+H]^+^; calcd. 456.257 (C_25_H_32_F_2_N_5_O). ^1^H-NMR (CDCl_3_, δ ppm, *J*, Hz): 8.12 (s, 1H, H-9), 7.75 (s, 1H, H-10), 7.51 (td, *J* = 9.0, 6.6, 1H, H-4), 7.16–7.09 (m, 4H, H-18, H-19, H-21, H-22), 6.79–6.73 (m, 2H, H-1, H-3), 4.51–4.44 (m, 2H, H-8), 3.44–3.32 (m, 2H, H-16), 3.04 (d, *J* = 13.3, 1H, H-11), 2.84 (sept, *J* = 6.8, 1H, H-23), 2.62 (d, *J* = 13.3, 1H, H-11′), 2.31 (m, 8H, H-12, H-13, H-14, H-15), 1.20 (d, *J* = 6.9, 6H, H-24, H-25). ^13^C-NMR (CDCl_3_, δC): 163.5, 163.4, 161.8, 161.7, 159.7, 159.6, 158.0, 158.0, (C-2 and C-6), 150.9 (C-10), 147.7 (C-17), 144.5 (C-9), 134.9 (C-20), 129.2, 129.19, 129.18, 129.1 (C-4), 129.0 (C-18, C-22), 126.25, 126.22 (C-5), 126.1 (C-19, C-21), 111.48, 111.46, 111.34, 111.32 (C-3), 104.3, 104.1, 104.0 (C-1), 71.7 (C-7), 65.4 (C-16), 62.11, 62.09 (C-11), 56.33, 56.30 (C-8), 54.2 (C-12, C-14), 52.9 (C-13, C-15), 33.6 (C-23), 23.9 (C-24, C-25).

*2-(2,4-Difluorophenyl)-1-(4-(((S)-4-(prop-1-en-2-yl)cyclohex-1-en-1-yl)methyl)piperazin-1-yl)-3-(1H-1,2,4-triazol-1-yl)propan-2-ol* **10b**. Yield 40%. Light yellow solid. Melting point: 92.8 °C with decomposition. HRMS: 458.274 [M+H]^+^; calcd. 458.274 (C_25_H_34_F_2_N_5_O). ^1^H-NMR (CDCl_3_, δ ppm, *J*, Hz): 8.12 (s, 1H, H-9), 7.75 (s, 1H, H-10), 7.51 (td, *J* = 9.0, 6.6, 1H, H-4), 6.82–6.71 (m, 2H, H-1, H-3), 5.54–5.47 (m, 1H, H-18), 4.70–4.61 (m, 2H, H-24), 4.47 (d, *J* = 2.4, 2H, H-8), 3.03 (dd, *J* = 13.5, 1.8, 1H, H-11), 2.80–2.64 (m, 2H, H-16), 2.61 (d, *J* = 13.6, 1H, H-11′), 2.39–2.11 (m, 8H, H-12, H-13, H-14, H-15), 2.10–1.80 (m, 5H, H-22, H-19, H-20), 1.75 (ddt, *J* = 12.6, 4.6, 2.5, 1H, H-21), 1.68 (s, 3H, H-25), 1.44–1.29 (m, 1H, H-21′). ^13^C-NMR (CDCl_3_, δC): 163.9, 163.8, 161.4, 161.3, 160.1, 160.0, 157.7, 157.5, (C-2 and C-6), 150.9 (C-10), 149.8 (C-17), 144.5 (C-9), 134.2 (C-23), 129.3, 129.19, 129.16, 129.10 (C-4), 126.3, 126.29, 126.20, 126.16 (C-5), 124.3 (C-18), 111.51, 111.48, 111.31, 111.27 (C-3), 108.4 (C-24), 104.4, 104.1, 103.9 (C-1), 71.59, 71.54 (C-7), 65.21, 65.12 (C-16), 62.08, 62.04 (C-11), 56.34, 56.29 (C-8), 54.3 (C-12, C-14), 52.9 (C-13, C-15), 41.07, 41.10 (C-20, C-20ds), 30.5 (C-19), 27.52, 27.55 (C-22, C-22ds), 27.40, 27.47 (C-21, C-21ds), 20.6 (C-25).

*2-(2,4-Difluorophenyl)-1-(4-(((1R,5S)-6,6-dimethylbicyclo[3.1.1]hept-2-en-2-yl)methyl)piperazin-1-yl)-3-(1H-1,2,4-triazol-1-yl)propan-2-ol* **10c**. Yield 50%. Light yellow solid. Melting point: 87.0 °C with decomposition. HRMS: 458.272 [M+H]^+^; calcd. 458.272 (C_25_H_34_F_2_N_5_O). ^1^H-NMR (CDCl_3_, δ ppm, *J*, Hz): 8.10 and 8.11: (s, 1H, H-9) and (s, 1H, H-9ds), 7.72 and 7.73: (s, 1H, H-10) and (s, 1H, H-10ds), 7.49 (td, *J* = 9.1, 8.6, 6.5, 1H, H-4), 6.75 (ddt, *J* = 11.4, 8.6, 2.3, 2H, H-1, H-3), 5.29–5.23 (m, 1H, H-18), 4.49–4.41 (m, 2H, H-8), 3.01 (ddd, *J* = 13.5, 4.7, 1.6, 1H, H-11), 2.81–2.71 (m, 1H, H-16), 2.69–2.57 (m, 2H, H-16′, H-11′), 2.35–2.10 (m, 11H, H-23′, H-12, H-13, H-14, H-15, H-19), 2.08 (td, *J* = 5.6, 1.4, 1H, H-22), 2.04–1.99 (m, 1H, H-20), 1.20 (d, *J* = 1.6, 3H, H-24), 1.01 and 0.98: (d, *J* = 8.6, 1H, H-23) and (d, *J* = 8.6, 1H, H-23ds), 0.73 and 0.72: (s, 3H, H-25) and (s, 3H, H-25ds). ^13^C-NMR (CDCl_3_, δC): 163.4, 163.3, 161.7, 161.6, 159.6, 159.6, 158.0, 157.9 (C-2 and C-6), 150.8 (C-10), 144.92, 144.87 (C-17, C-17ds), 144.48, 144.46 (C-9, C-9ds), 129.17, 129.13, 129.11, 129.07 (C-4), 126.26, 126.24, 126.17 (C-5), 119.83, 119.89 (C-18), 111.4, 111.3 (C-3), 104.3, 104.1, 103.9 (C-1), 71.5, 71.6, 71.5 (C-7, C-7ds), 64.09, 64.04 (C-16), 62.06, 62.03, 62.01, 61.98 (C-11, C-11ds), 56.31, 56.28, 56.25 (C-8, C-8ds), 54.2 (C-12, C-14), 53.1 (C-13, C-15), 44.1, 44.2 (C-22, C-22ds), 40.7 (C-20), 37.74, 37.75 (C-21, C-21ds), 31.50, 31.52 (C-23, C-23ds), 31.1 (C-19), 26.0 (C-24, C-24ds), 20.83, 20.87 (C-25, C-25ds).

*2-(2,4-Difluorophenyl)-1-(4-(2-((1R,5S)-6,6-dimethylbicyclo[3.1.1]hept-2-en-2-yl)ethyl)piperazin-1-yl)-3-(1H-1,2,4-triazol-1-yl)propan-2-ol* **10d**. Yield 40%. Light yellow semi-solid. HRMS: 472.289 [M+H]^+^; calcd. 472.289 (C_26_H_36_F_2_N_5_O). ^1^H-NMR (CDCl_3_, δ ppm, *J*, Hz): 8.10 (d, *J* = 2.6, 1H, H-9), 7.73 (d, *J* = 1.4, 1H, H-10), 7.54–7.44 (m, 1H, H-4), 6.80–6.71 (m, 2H, H-1, H-3), 5.16–5.11 (m, 1H, H-19), 4.51–4.40 (m, 2H, H-8), 3.01 (ddd, *J* = 13.5, 4.7, 1.6, 1H, H-11), 2.60 (d, *J* = 13.6, 1H, H-11′), 2.41–1.97 (m, 16H, H-12, H-13, H-14, H-15, H-16, H-17, H-20, H-21, H-24′), 1.92 (td, *J* = 5.6, 1.5, 1H, H-23), 1.20 (s, 3H, H-25), 1.06 (d, *J* = 8.5, 1H, H-24′), 0.74 (s, 3H, H-26). ^13^C-NMR (CDCl_3_, δC): 163.8, 163.7, 161.4, 161.2, 160.1, 159.9, 157.6, 157.5 (C-2 and C-6), 150.8 (C-10), 146.0 (C-18), 144.5 (C-9), 129.20, 129.14, 129.11, 129.05 (C-4), 126.19, 126.15, 126.06, 126.02 (C-5), 117.0 (C-19), 111.4, 111.2 (C-3), 104.4, 104.1, 103.9 (C-1), 71.68, 71.63 (C-7), 62.08, 62.04 (C-11), 56.4 (C-16), 56.22, 56.17 (C-8), 54.2 (C-12, C-14), 52.9 (C-13, C-15), 45.7 (C-23), 40.6 (C-21), 37.8 (C-22), 34.1 (C-17), 31.5 (C-24), 31.1 (C-20), 26.1 (C-25), 21.1 (C-26).

*(E)-2-(2,4-Difluorophenyl)-1-(4-(3,7-dimethylocta-2,6-dien-1-yl)piperazin-1-yl)-3-(1H-1,2,4-triazol-1-yl)propan-2-ol* **10e**. Yield 68%. Yellow oil. HRMS: 460.289 [M+H]^+^; calcd. 460.289 (C_25_H_36_F_2_N_5_O). ^1^H-NMR (CDCl_3_, δ ppm, *J*, Hz): 8.10 (s, 1H, H-9), 7.73 (s, 1H, H-10), 7.49 (td, *J* = 9.0, 6.6, 1H, H-4), 6.79–6.71 (m, 2H, H-1, H-3), 5.15–5.08 (m, 1H, H-17), 5.01–4.95 (m, 1H, H-21), 4.50–4.41 (m, 2H, H-8), 3.02 (dd, *J* = 13.5, 1.7, 1H, H-11), 2.90–2.80 (m, 2H, H-16), 2.61 (d, *J* = 13.6, 1H, H-11′), 2.43–2.12 (m, 8H, H-12, H-13, H-14, H-15), 2.06–1.87 (m, 4H, H-19, H-20), 1.58 (s, 3H, H-23), 1.55 (s, 3H, H-24), 1.52 (s, 3H, H-25). ^13^C-NMR (CDCl_3_, δC): 163.5, 163.4, 161.5, 161.4, 159.8, 159.7, 157.8, 157.7 (C-2 and C-6), 150.8 (C-10), 144.4 (C-9), 139.0 (C-18), 131.3 (C-22), 129.12, 129.08, 129.05, 129.00 (C-4), 126.12, 126.09, 126.01, 125.99 (C-5), 123.8 (C-21), 120.1 (C-17), 111.40, 111.38, 111.24, 111.21 (C-3), 104.3, 104.1, 103.9 (C-1), 71.60, 71.56 (C-7), 62.00, 61.97 (C-11), 56.2 (C-16), 56.17, 56.13 (C-8), 55.5 (C-12, C-14), 52.8 (C-13, C-15), 39.5 (C-19), 26.1 (C-20), 25.5 (C-23), 17.5 (C-25), 16.1 (C-24).

*2-(2,4-Difluorophenyl)-1-(4-(3,7-dimethyloct-6-en-1-yl)piperazin-1-yl)-3-(1H-1,2,4-triazol-1-yl)propan-2-ol* **10f**. Yield 30%. Yellow oil. HRMS: 462.304 [M+H]^+^; calcd. 462.304 (C_25_H_38_F_2_N_5_O). ^1^H-NMR (CDCl_3_, δ ppm, *J*, Hz): 8.12 (s, 1H, H-9), 7.75 (s, 1H, H-10), 7.51 (td, *J* = 9.0, 6.6, 1H, H-4), 6.87–6.67 (m, 2H, H-1, H-3), 5.29 (s, 1H, OH), 5.04 (dt, *J* = 8.8, 4.1, 1H, H-21), 4.57–4.38 (m, 2H, H-8), 3.03 (dd, *J* = 13.5, 1.7, 1H, H-11), 2.62 (d, *J* = 13.6, 1H, H-11′), 2.47–2.13 (m, 10H, H-12, H-13, H-14, H-15, H-16), 1.98–1.82 (m, *J* = 6.9, 2H, H-20), 1.63 (s, 3H, H-23), 1.55 (s, 3H, H-24), 1.47–1.04 (m, 5H, H-17, H-18, H-19), 0.82 (d, *J* = 6.3, 3H, H-25). ^13^C-NMR (CDCl_3_, δC): 163.9, 163.7, 161.4, 161.3, 160.1, 160.0, 157.6, 157.5 (C-2 and C-6), 150.9 (C-10), 144.5 (C-9), 131.1 (C-22), 129.22, 129.16, 129.13, 129.07 (C-4), 126.23, 126.20, 126.10, 126.07 (C-5), 124.6 (C-21), 111.52, 111.48, 111.31, 111.28 (C-3), 104.4, 104.2, 103.9 (C-1), 71.64, 71.58 (C-7), 62.08, 62.04 (C-11), 56.5 (C-16), 56.26, 56.21 (C-8), 54.2 (C-12, C-14), 53.2 (C-13, C-15), 37.0 (C-19), 33.6 (C-17), 30.9 (C-18), 25.6 (C-23), 25.3 (C-20), 19.5 (C-25), 17.5 (C-24).

*2-(2,4-Difluorophenyl)-1-(4-(3,7-dimethyloctyl)piperazin-1-yl)-3-(1H-1,2,4-triazol-1-yl)propan-2-ol* **10g**. Yield 30%. Yellow oil. HRMS: 464.321 [M+H]^+^; calcd. 464.321 (C_25_H_40_F_2_N_5_O). ^1^H-NMR (CDCl_3_, δ ppm, *J*, Hz): 8.12 (s, 1H, H-9), 7.75 (s, 1H, H-10), 7.56–7.46 (m, 1H, H-4), 6.81–6.73 (m, 2H, H-1, H-3), 4.53–4.43 (m, 2H, H-8), 3.03 (dd, *J* = 13.6, 1.7, 1H, H-11), 2.62 (d, *J* = 13.6, 1H, H-11′), 2.48–2.16 (m, 10H, H-12, H-13, H-14, H-15, H-16), 1.53–1.29 (m, 3H, H-18, H-19, H-22), 1.28–0.99 (m, 7H, H-19′, H-17, H-20, H-21), 0.81 (d, *J* = 6.6, 6H, H-23, H-24), 0.80 (d, *J* = 6.5, 3H, H-25). ^13^C-NMR (CDCl_3_, δC): 163.9, 163.8, 161.4, 161.3, 160.1, 160.0, 157.7, 157.5 (C-2 and C-6), 150.9 (C-10), 144.5 (C-9), 129.25, 129.19, 129.15, 129.09 (C-4), 126.25, 126.21, 126.12, 126.08 (C-5), 111.52, 111.49, 111.32, 111.29 (C-3), 104.4, 104.2, 103.9 (C-1), 71.72, 71.67 (C-7), 62.14, 62.10 (C-11), 56.5 (C-16), 56.30, 56.25 (C-8), 54.2 (C-12, C-14), 53.2 (C-13, C-15), 39.1 (C-21), 37.2 (C-20), 33.7 (C-19), 31.3 (C-18), 27.8 (C-22), 24.5 (C-17), 22.6 (C-24), 22.5 (C-23), 19.66, 19.64 (C-25 and C-25ds).

*2-(2,4-Difluorophenyl)-1-(4-phenylpiperazin-1-yl)-3-(1H-1,2,4-triazol-1-yl)propan-2-ol* **10h**. Yield 35%. Brown oil. HRMS: 400.1944 [M+H]^+^; calcd. 400.1949 (C_21_H_24_F_2_N_5_O). ^1^H-NMR (CDCl_3_, δ ppm, *J*, Hz): 8.18 (s, 1H, H-9), 7.82 (s, 1H, H-10), 7.62–7.57 (m, 1H, H-4), 7.23–7.18 (m, 2H, H-18, H-20), 6.85–6.77 (m, 5H, H-1, H-3, H-17, H-19, H-21), 5.19 (br.s, 1H, OH), 4.57–4.47 (m, 2H, H-8), 3.12 (d, *J* = 12.6, 1H, H-11), 2.70 (d, *J* = 13.1, 1H, H-11′), 3.03 (br.s, 4H, H-13, H-15), 2.49 (br.s, 4H, H-12, H-14). ^13^C-NMR (CDCl_3_, δC): 163.9, 163.8, 161.9, 161.8, 160.0, 159.9, 158.0, 157.9 (C-2 and C-6), 151.2 (C-10), 150.9 (C-16), 144.7 (C-9), 129.46, 129.41, 129.34, 129.30 (C-4), 129.2 (C-18, C-20), 126.1 (C-5), 120.1 (C-19), 116.1 (C-17, C-21), 111.8, 111.6 (C-3), 104.6, 104.4, 104.2 (C-1), 72.18, 72.14 (C-7), 62.36, 62.33 (C-11), 56.29, 56.26 (C-8), 54.4 (C-13, C-15), 49.2 (C-12, C-14).

### 3.2. Biology

#### 3.2.1. Microdilution Test

Minimum inhibitory concentrations of the compounds were determined according to European Committee on Antimicrobial Susceptibility Testing (EUCAST) guidelines [45]. Briefly, solutions of the compounds at desired concentrations were prepared in DMSO. Serial two-fold dilutions were obtained in cell culture-treated 96 well flat bottom microplates (Greiner Bio One International, Kremsmünster, Austria) using RPMI 1640 medium (Sigma-Aldrich, St. Louis, MO, USA) with l-glutamine, without sodium bicarbonate and 2% glucose, which were stored at −70 °C until used. For the test, the microplates were allowed to reach room temperature before inoculum (1–5 × 10^5^ cfu/mL) was added, then they were incubated at 36 °C for 24 h. The absorbances of the wells were measured at 530 nm using a spectrophotometer (BioTek Instruments, Vinooski, VT, USA). MIC values were determined as ≥50% reduction in growth according to drug-free control. The activity of the compounds was tested at least twice against standard strains (*C. krusei* ATCC 6258, *C. parapsilosis* ATCC 22019, *C. parapsilosis* ATCC 90018, *C. glabrata* ATCC 90030, *C. albicans* ATCC 90028, *C. albicans* ATCC 64547, *C. tropicalis* ATCC 750) and clinical isolates (seven fluconazole-resistant *C. parapsilosis* and four fluconazole susceptible-increased exposure/resistant *C. glabrata*) using fluconazole as positive control. The results are presented as ranges of MIC values from the repeated tests.

#### 3.2.2. Cytotoxicity Test

Cytotoxic effects of the compounds were tested on murine fibroblast (3T3) cells according to the MTT (3-(4,5-dimethylthiazol-2-yl)-2,5-diphenyltetrazolium bromide) assay [46]. The cells were grown in Dulbecco’s modified Eagle’s medium (DMEM), supplemented with 10% FBS at 37 °C and humidified atmosphere containing 5% CO_2_, and seeded at 10,000 cells/well density in 96-well plates and incubated for an additional 24 h. Following the treatment with the title azoles (**10a**, **10d**–**h**) at varying concentrations between 0.016–2 µg/mL and incubation for 24 and 48 h, the cell medium containing the compounds was removed and replaced with 1 mg/mL MTT solution in 100 µL culture medium. The cells were further incubated for 3 h under the same conditions. The MTT solutions were removed, and 100 µL DMSO was added to the wells to dissolve the formazan crystals, followed by optic density measurements at 570 nm using a microplate reader (SpectraMax M2 Molecular Devices Limited, Berkshire, UK). The cytotoxicity of the compounds was determined at 24 and 48 h by calculating the cell viability percentage of the treated cells with respect to the untreated (control) cells, which were considered 100% viable. Each experiment was performed in duplicate, and Student’s t-test was applied for statistical analysis.

### 3.3. Molecular Modeling Study

The title compounds were modelled and optimized using LigPrep (2021-4, Schrödinger LLC, New York, NY, USA) and MacroModel (2021-4, Schrödinger LLC, New York, NY, USA) according to the OPLS4 (2021-4, Schrödinger LLC, New York, NY, USA) forcefield parameters [47]. Molecular descriptors were computed using the SwissADME web server (www.swissadme.ch, accessed on 1 April 2023) [39]. Gasteiger charges were added to the ligand atoms, and the ligands were converted to pdbqt format using OpenBabel (The Open Babel Package, version 2.3.1 http://openbabel.org, accessed on 1 April 2023) [48]. The crystallographic structure of *C. albicans* CYP51 (PDB ID: 5TZ1 [31], resolution: 2.00 Å) was downloaded from the RCSB protein data bank (www.rcsb.org, accessed on 1 April 2023) [49] and prepared for docking using the Protein Preparation Wizard of Maestro (2021-4, Schrödinger LLC, New York, NY, USA) [50] to remove the redundant molecules add H atoms, assign bond orders, generate ionization and tautomeric states, and assign H bonds. Gasteiger charges were added to the protein atoms, and the protein structure was converted to pdbqt format using AutoDockTools. Grid maps were generated for the ligand and receptor atoms for the active site defined as a cube of ~8000 Å^3^ centered on the coordinates x = 70.49 y = 65.24 z = 4.453 using AutoGrid (v4, The Scripps Research Institute, San Diego, CA, USA). Molecular docking was performed using AutoDock (v4.2.6, The Scripps Research Institute, San Diego, CA, USA) according to Lamarckian Genetic Algorithm with 2,500,000 maximum energy evaluations per run and 50 runs per ligand [51]. The results were visually evaluated, and the images were generated using Maestro and Gimp (v2.10.14, The GIMP Developer Team, www.gimp.org, accessed on 1 April 2023).

### 3.4. X-ray Diffraction Analysis

The X-ray diffraction experiment was carried out at 296(2) K on a Bruker KAPPA APEX II diffractometer (graphite-monochromated Mo Kα radiation). Reflection intensities were corrected for absorption by the SADABS-2016 program [52]. The structure of compounds was solved by direct methods using the SHELXT-2014 program [53] and refined by anisotropic (isotropic for all H atoms) full-matrix least-squares method against *F^2^* of all reflections by SHELXL-2018 [54]. The positions of the hydrogen atoms were calculated geometrically and refined in the riding model.

Crystallographic data for **10b** have been deposited at the Cambridge Crystallographic Data Centre as supplementary publication no. CCDC 2254411. Copy of the data can be obtained, free of charge, on application to CCDC, 12 Union Road, Cambridge CB21EZ, UK (Fax: +44-122-3336033 or e-mail: deposit@ccdc.cam.ac.uk; internet: www.ccdc.cam.ac.uk, accessed on 1 April 2023).

## 4. Conclusions

In summary, we have synthesized novel monoterpene-containing azoles by combining azole core and monoterpene moiety through a piperazine linker. All synthesized compounds showed excellent antifungal activity against both azole-susceptible and azole-resistant strains of *Candida* spp. Compounds **10a** and **10c** also exhibited superior activity compared to the control drug fluconazole against clinical isolates, including *Candida parapsilosis* and *Candida glabrata*. Additionally, synthesized products showed low cytotoxicity in the MTT test.

## Figures and Tables

**Table 1:** MIC values (μg/mL) of the azoles **10a–h** obtained using EUCAST methodology.

Compound	*Candida albicans* ATCC 64547	*Candida albicans* ATCC 90028	*Candida glabrata* ATCC 90030	*Candida krusei* ^1^ ATCC 6258	*Candida parapsilosis* ATCC 22019	*Candida parapsilosis* ATCC 90018	*Candida tropicalis* ATCC 750
**10a**	<0.004	<0.004	0.5	0.25–0.5	0.016–0.03	<0.004	<0.004–0.008
**10b** ^2^	n.t.	0.031	n.t.	0.5	n.t.	0.031	n.t.
**10c**	0.03	0.016–0.03	0.5–2	0.5–1	0.06	0.008	0.016–0.03
**10d**	0.004–0.016	0.008–0.03	0.5–2	0.25–0.5	0.06–0.125	0.008–0.03	0.016–0.03
**10e**	0.016–0.06	0.016–0.06	1–4	1	0.06–0.25	0.06	0.06–0.25
**10f**	0.016–0.03	0.016–0.06	0.5–2	1–2	0.125–0.25	0.06–0.125	0.06–0.25
**10g**	0.08–0.03	0.016–0.03	0.25–1	1–2	0.06–0.25	0.03–0.06	0.016–0.06
**10h**	<0.004–0.008	<0.004–0.008	0.25–0.05	1	0.06–0.125	0.016–0.03	0.016–0.03
FCZ	0.25–0.5	<0.125	2–4	16–32	1–2	0.5–1	0.5–1

n.t.—not tested. ^1^ Fluconazole resistant strain. ^2^ Compound **10b** was tested using CLSI methodology [36].

**Table 2:** MIC values (μg/mL) of the azoles **10a**, **10c**, and **10h** against clinical isolates according to EUCAST methodology.

Clinical Isolates	Species	10a	10c	10h	FCZ
15666	*C. parapsilosis*	0.5	1–2	16	64
15793	*C. parapsilosis*	0.25	0.25–0.5	1	16
16120	*C. parapsilosis*	1–2	2–4	4–16	64
16393	*C. parapsilosis*	0.25–0.5	2	8	64
16647	*C. parapsilosis*	0.25	1–2	8–16	32
16681	*C. parapsilosis*	0.25	1–2	16	32
16730	*C. parapsilosis*	0.25–1	2	8–16	32
19823	*C. glabrata*	4	8–16	2	64
21743	*C. glabrata*	0.25	0.25–1	0.125–0.25	2
25589	*C. glabrata*	0.5	0.5–2	0.125–0.5	2–8
25878	*C. glabrata*	0.5–1	4	0.25–0.5	16–32

**Table 3:** Docking scores (kcal/mol).

Compound	Docking Score	Compound	Docking Score
**10a**	−7.5	**10f**	−9.0
**10b**	−8.7	**10g**	−8.2
**10c**	−8.5	**10h**	−9.2
**10d**	−9.1	Fluconazole	−6.5
**10e**	−9.4		

## Data Availability

The data presented in this study are available on request from the corresponding author.

## Supplementary Materials

The following supporting information can be downloaded at: https://www.mdpi.com/article/10.3390/antibiotics12050818/s1, Figures S1–S24 (p. 2–25)—copies of NMR spectra of compounds **9b, c, g, d** and **10a–h**; Figures S25–S31 (p. 26–32)—HRMS of compounds **10a–g**; Table S1–S3 (p. 33)—cytotoxicity results; Table S4–S6 (p. 34)—molecular modeling.
