# Supplementary material for: Rational Design of New Monoterpene-Containing Azoles and Their Antifungal Activity
Source: Antibiotics (Basel). 2023 Apr 27;12(5):818. doi: 10.3390/antibiotics12050818 (PMC10215469; doi:10.3390/antibiotics12050818)
Supplement: Supplementary file 1 [file antibiotics-12-00818-s001.zip › antibiotics-2365487-supplementary.pdf]

Supplementary Information

Rational design of new monoterpene-containing azoles and their antifungal activity

Nikolai S. Li-Zhulanov [1,*], Nadezhda P. Zaikova [1], Suat Sari [2], Dolunay Gülmez [3], Suna Sabuncuoğlu [4], Keriman Ozadali-Sari [2], Sevtap Arikan-Akdagli [3], Andrey A. Nefedov [1], Tatyana V. Rybalova [1], Konstantin P. Volcho [1,*], Nariman F. Salakhutdinov [1]

[1] N.N. Vorozhtsov Novosibirsk Institute of Organic Chemistry SB RAS, 9 Akademika Lavrentieva Ave., Novosibirsk, 630090, Russia

[2] Hacettepe University Faculty of Pharmacy, Dept. of Pharmaceutical Chemistry, Sihhiye Ankara, 06100, Turkey

[3] Hacettepe University Faculty of Medicine, Dept. of Medical Microbiology, Sihhiye Ankara, 06100, Turkey

[4] Hacettepe University Faculty of Pharmacy, Dept. of Pharmaceutical Toxicology, Sihhiye Ankara, 06100, Turkey

* Correspondence: lizhulan@nioch.nsc.ru (N.S.L.-Z.), volcho@nioch.nsc.ru (K.P.V.); Tel.: +7 (383) 330-88-70

1. NMR ^{1}H and ^{13}C spectra of the compounds **9b, c, g, d** and **10a-h** (p. 2-25).

2. HRMS of the compounds **10a-g** (p.26-32)

3. Cytotoxicity results (p. 33).

4. Molecular modeling (p. 34).

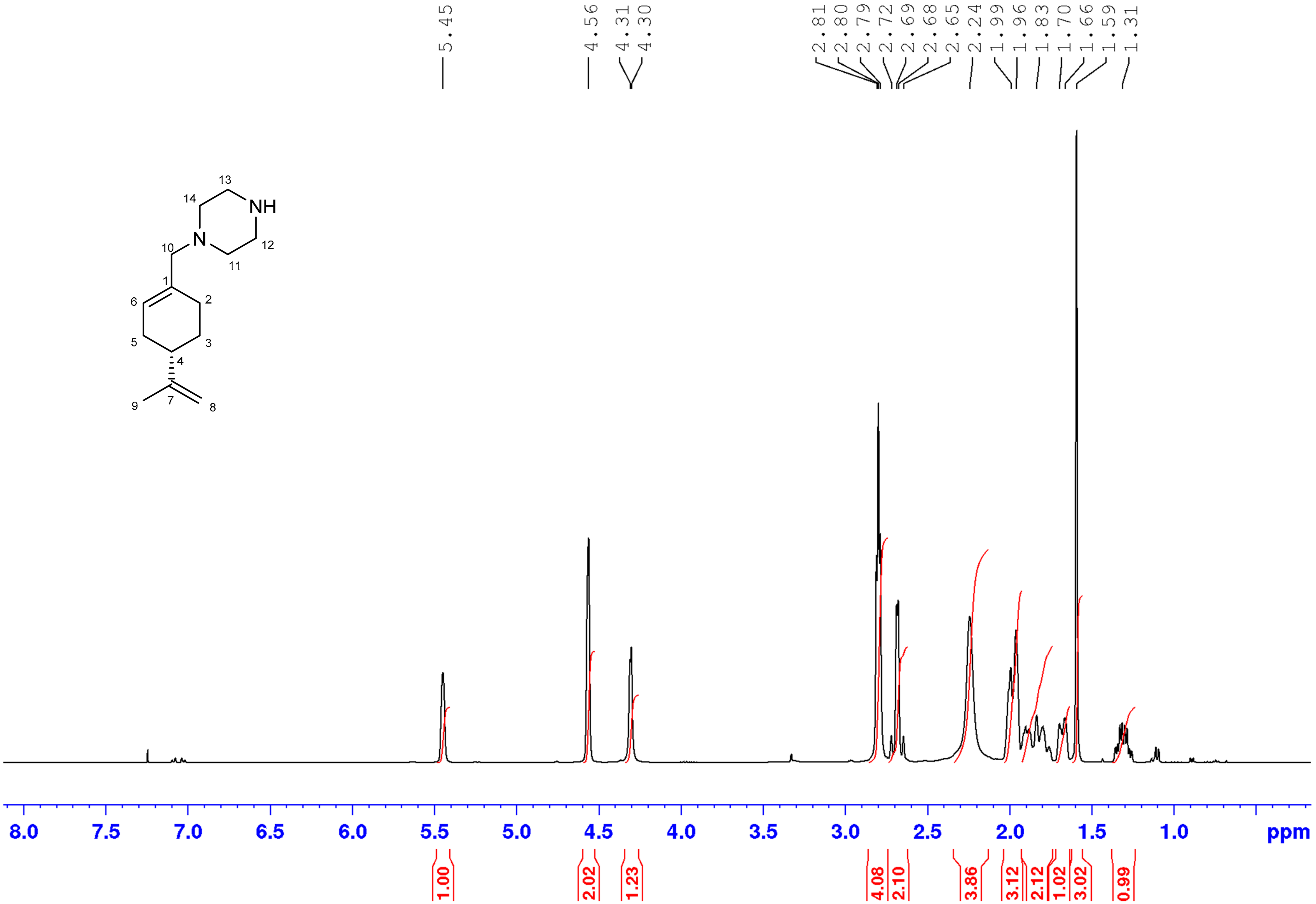

Figure S1. ^{1}H-NMR spectroscopic data for compound **9b**.

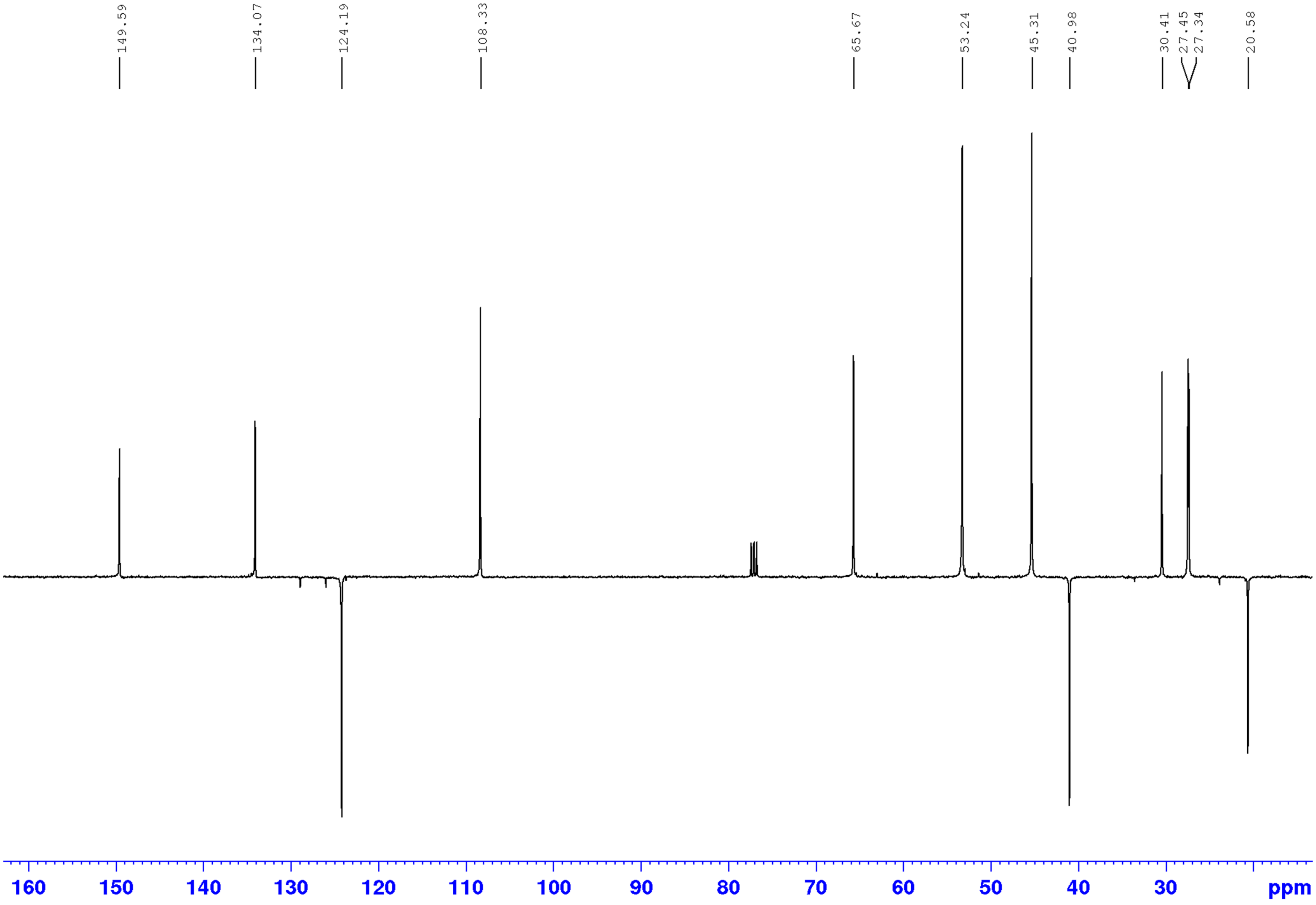

Figure S2. ^{13}C-NMR spectroscopic data for compound **9b**.

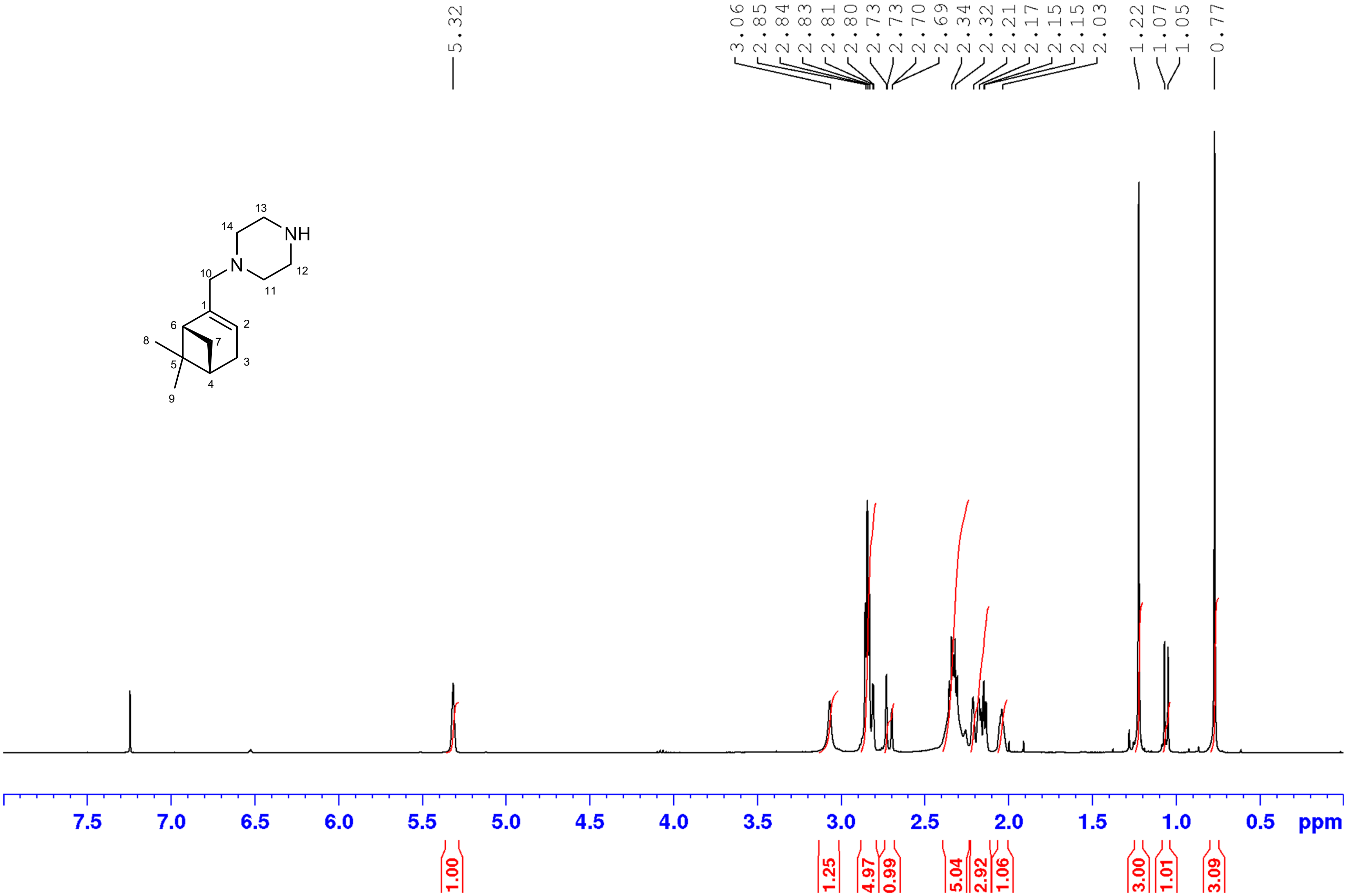

Figure S3. ^{1}H-NMR spectroscopic data for compound **9c**.

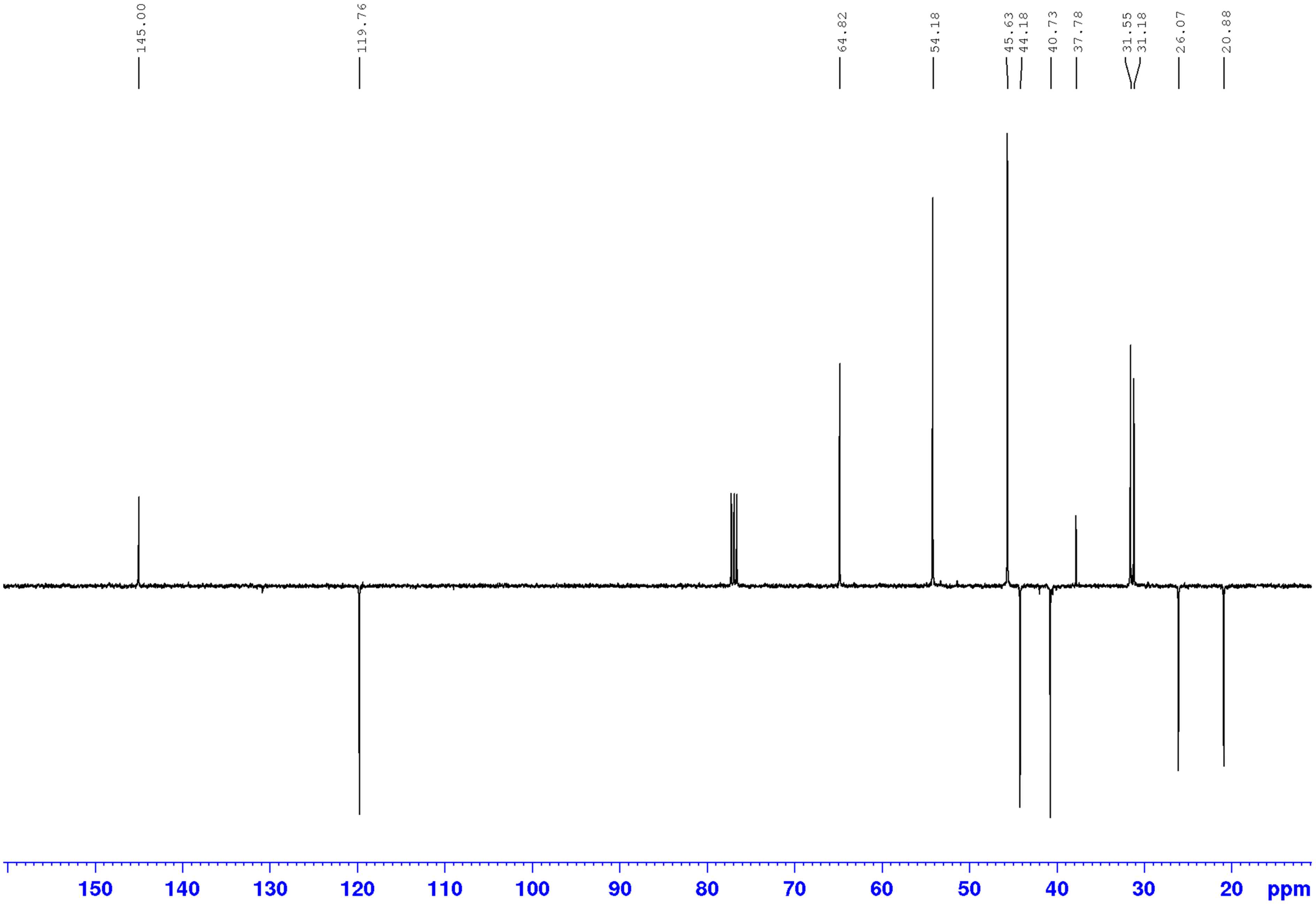

Figure S4. ^{13}C-NMR spectroscopic data for compound **9c**.

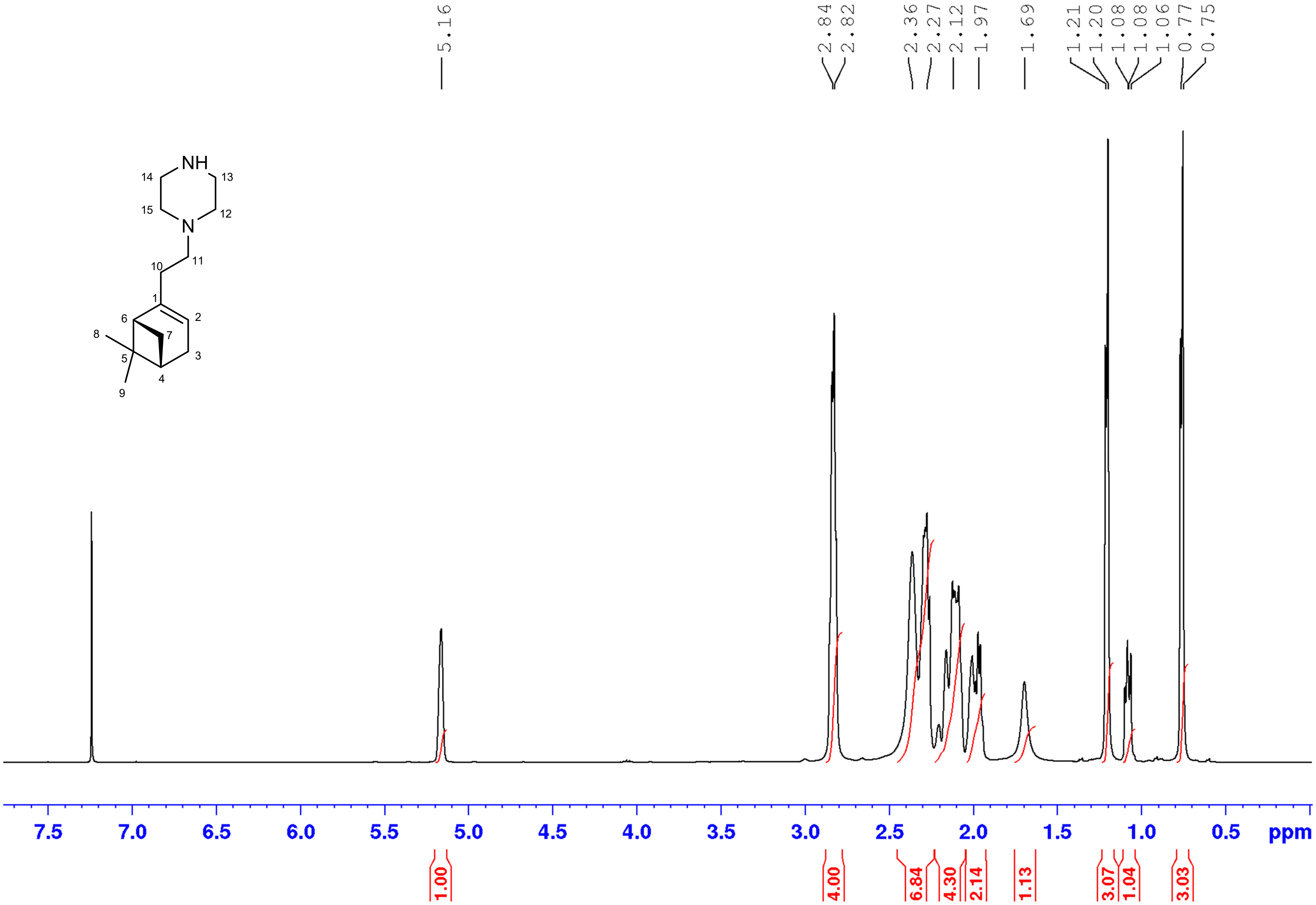

Figure S5. ^{1}H-NMR spectroscopic data for compound **9d**.

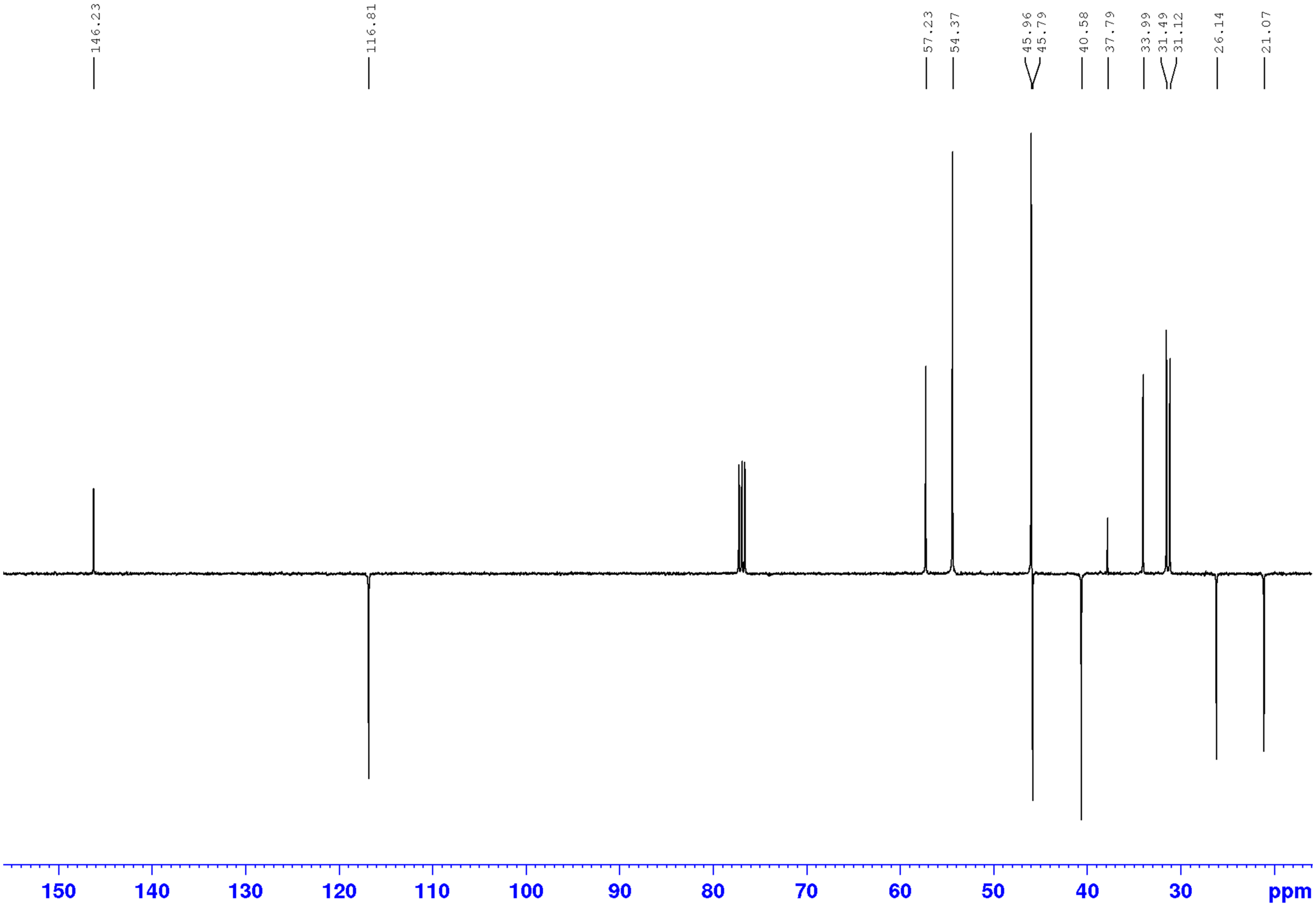

Figure S6. ^{13}C -NMR spectroscopic data for compound **9d**.

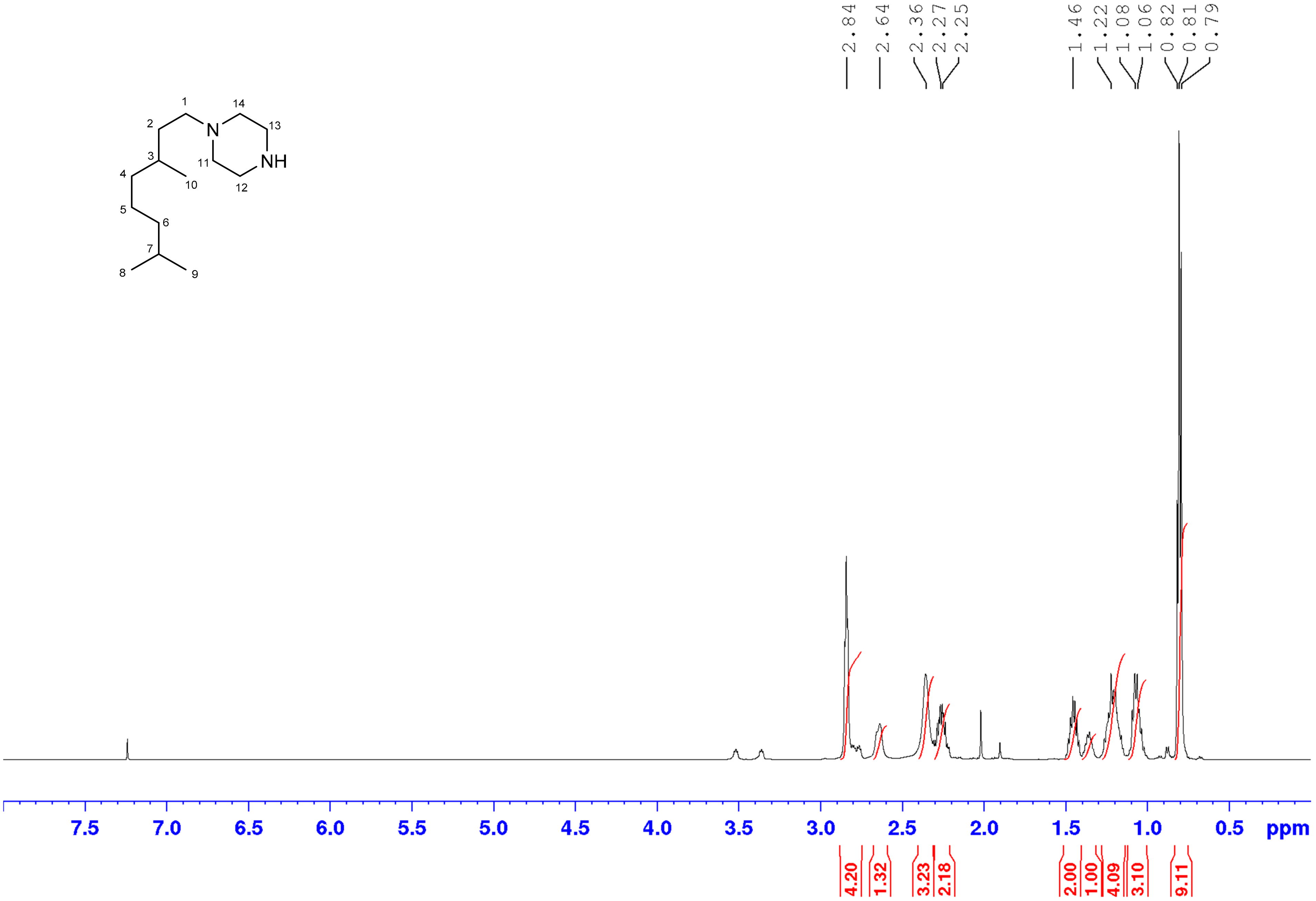

Figure S7. ^{1}H-NMR spectroscopic data for compound **9g**.

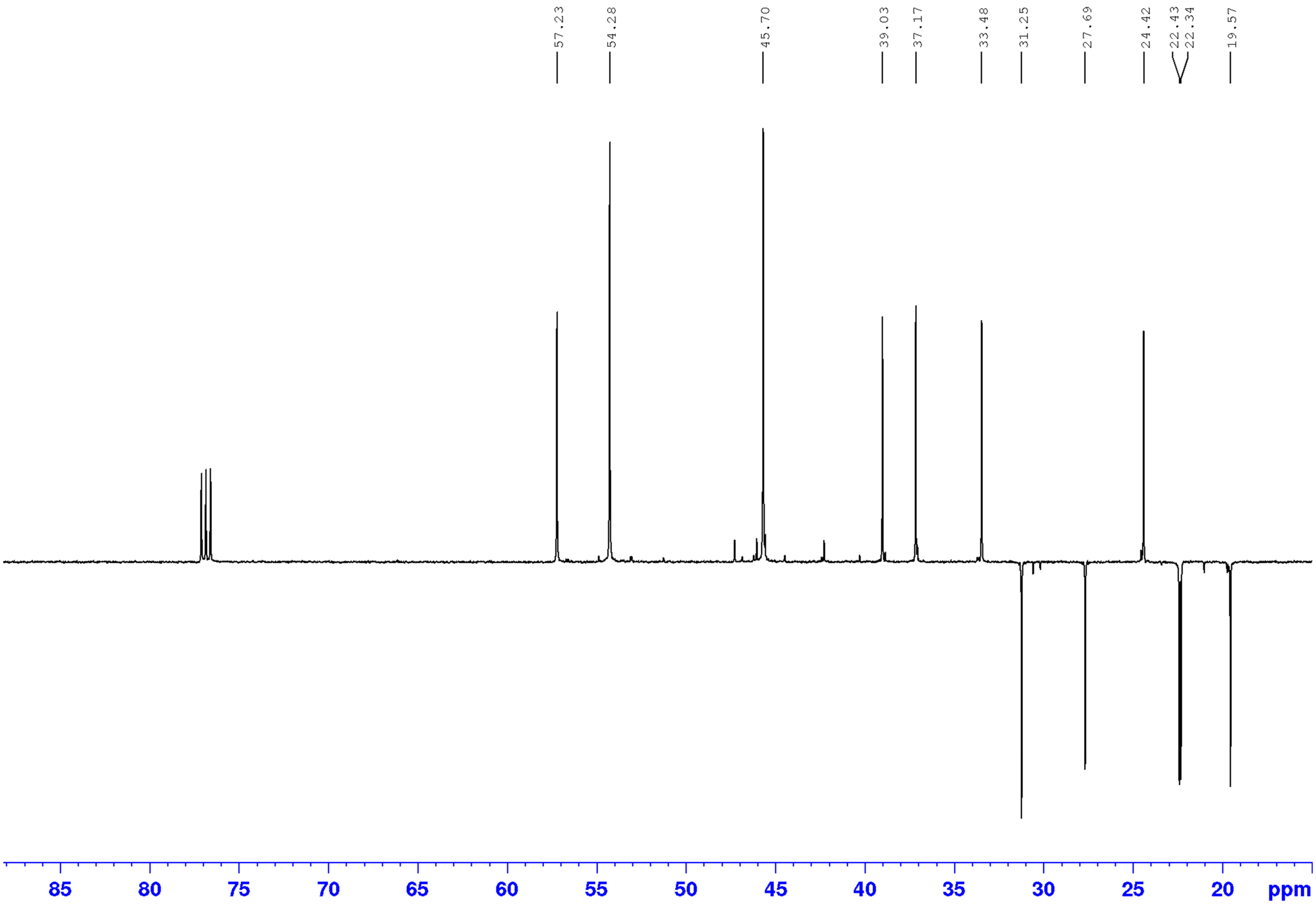

Figure S8. ^{13}C-NMR spectroscopic data for compound **9g**.

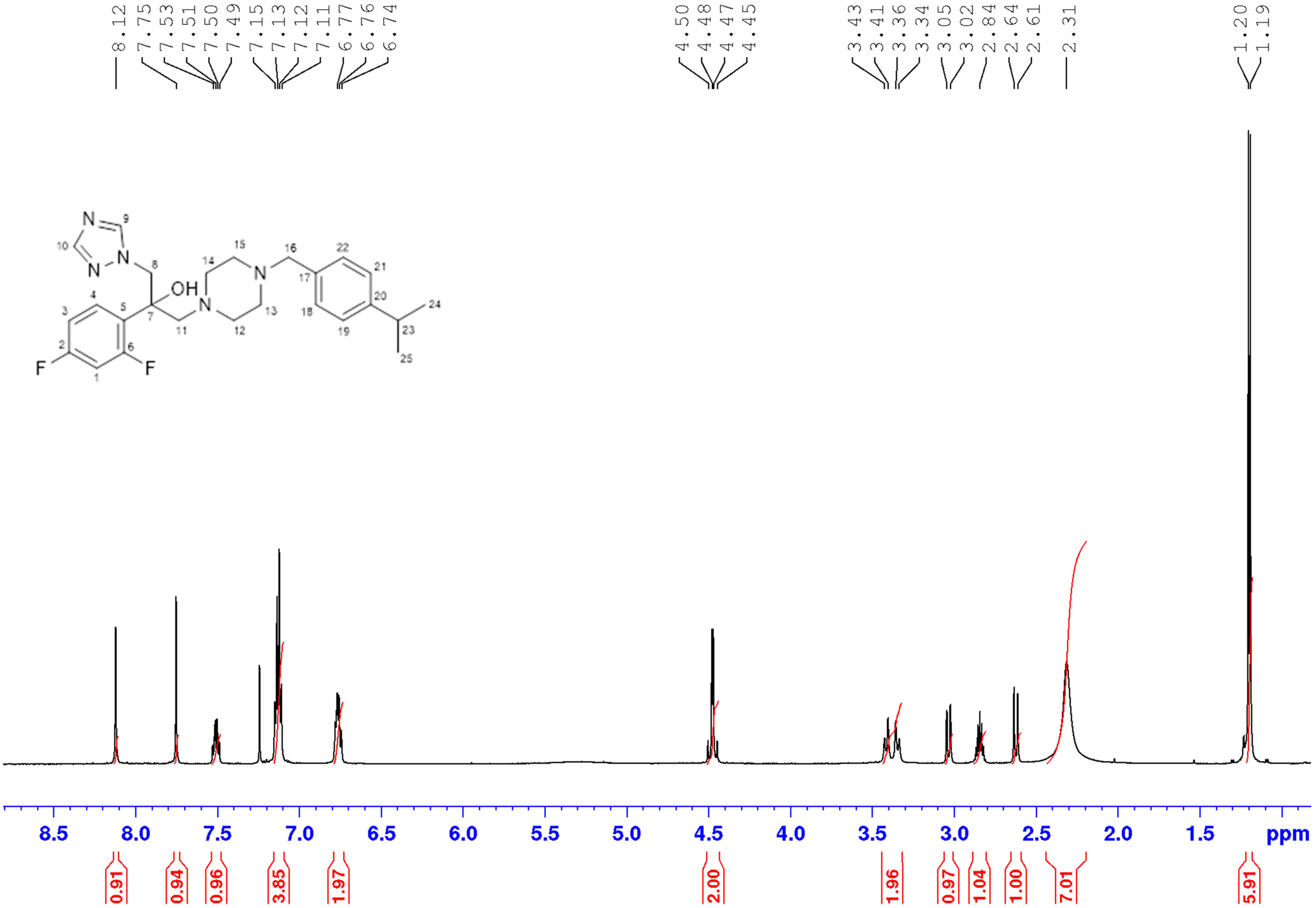

Figure S9. ^{1}H-NMR spectroscopic data for compound **10a**.

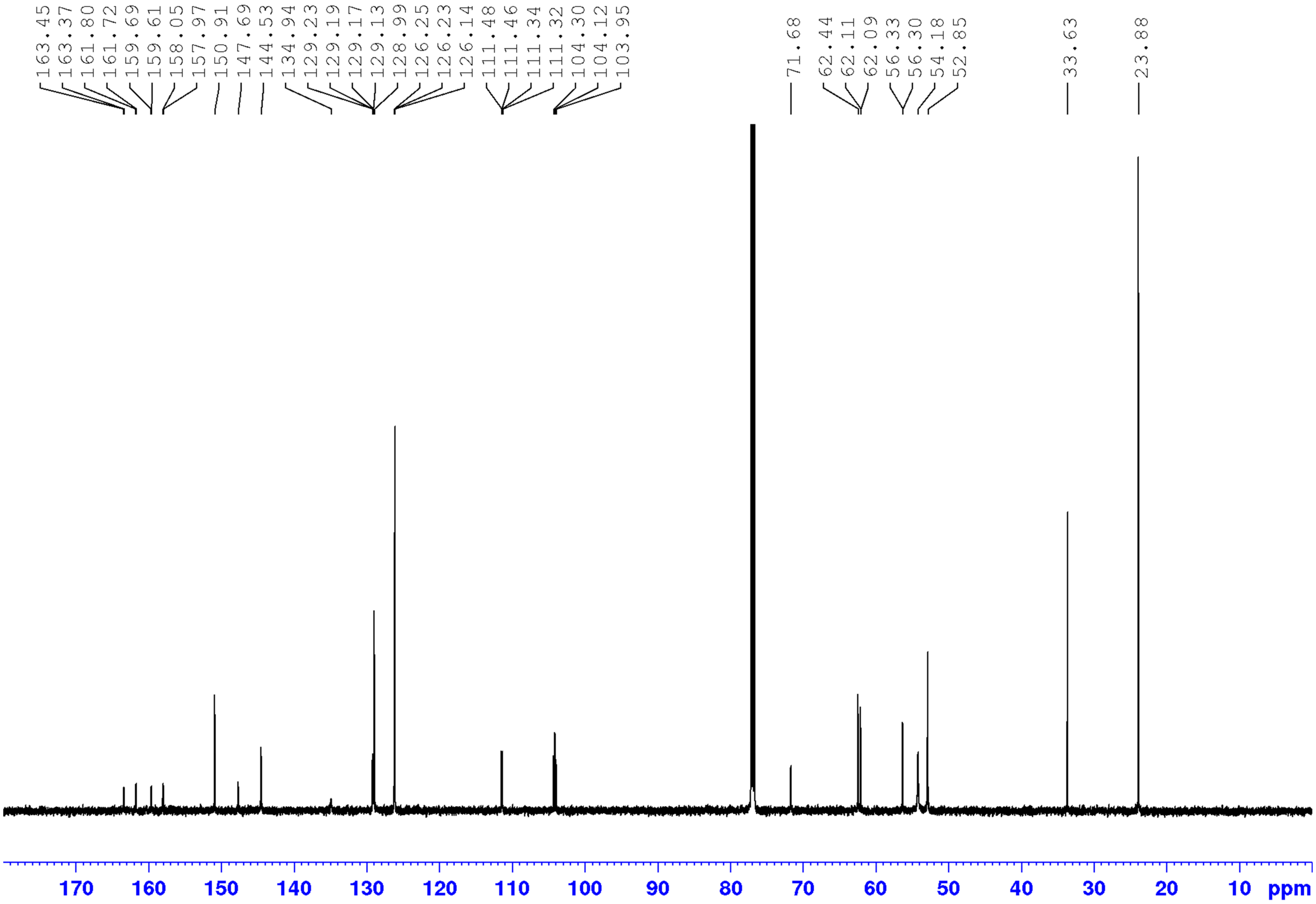

Figure S10. ^{13}C-NMR spectroscopic data for compound **10a**.

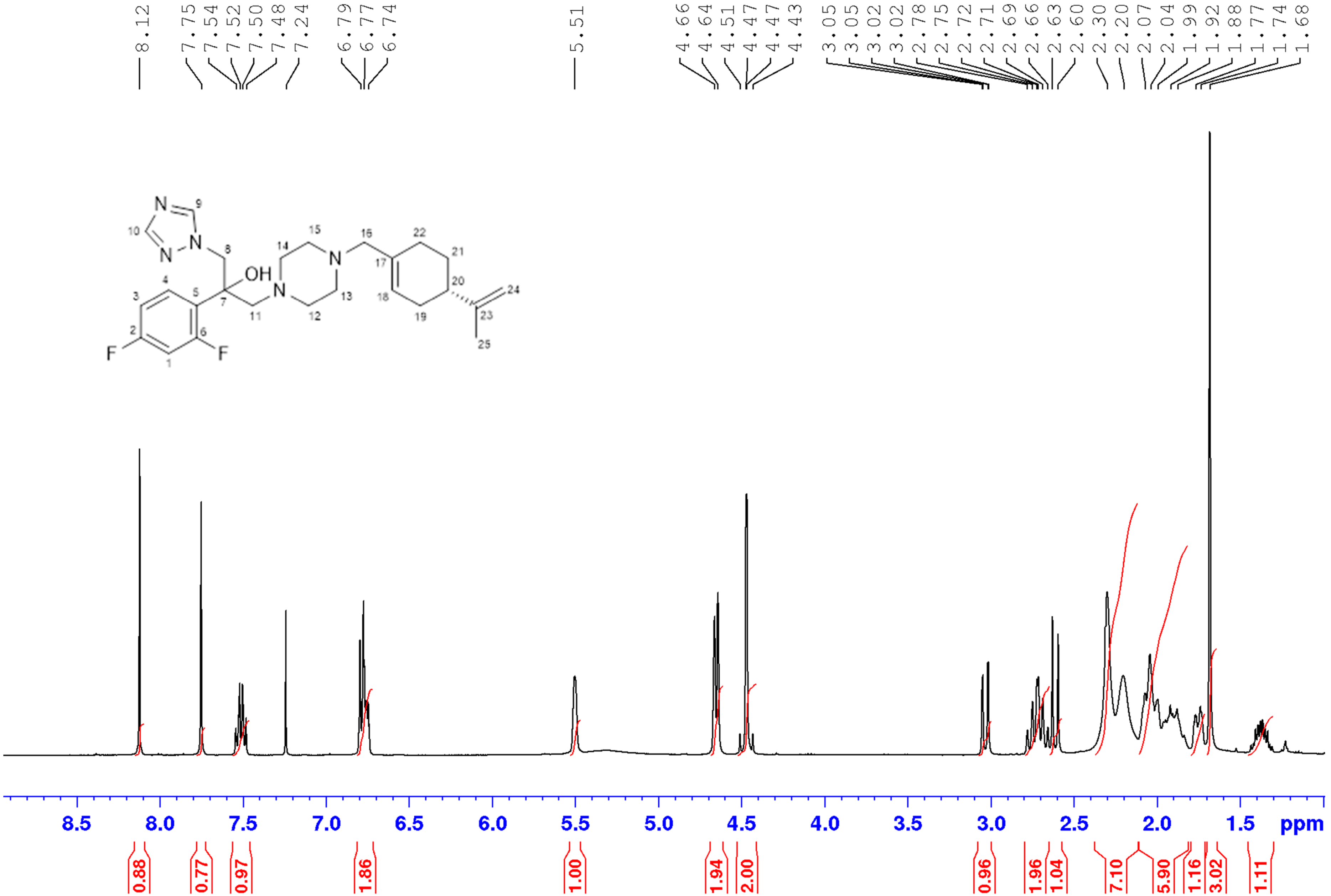

Figure S11. ^{1}H-NMR spectroscopic data for compound **10b**.

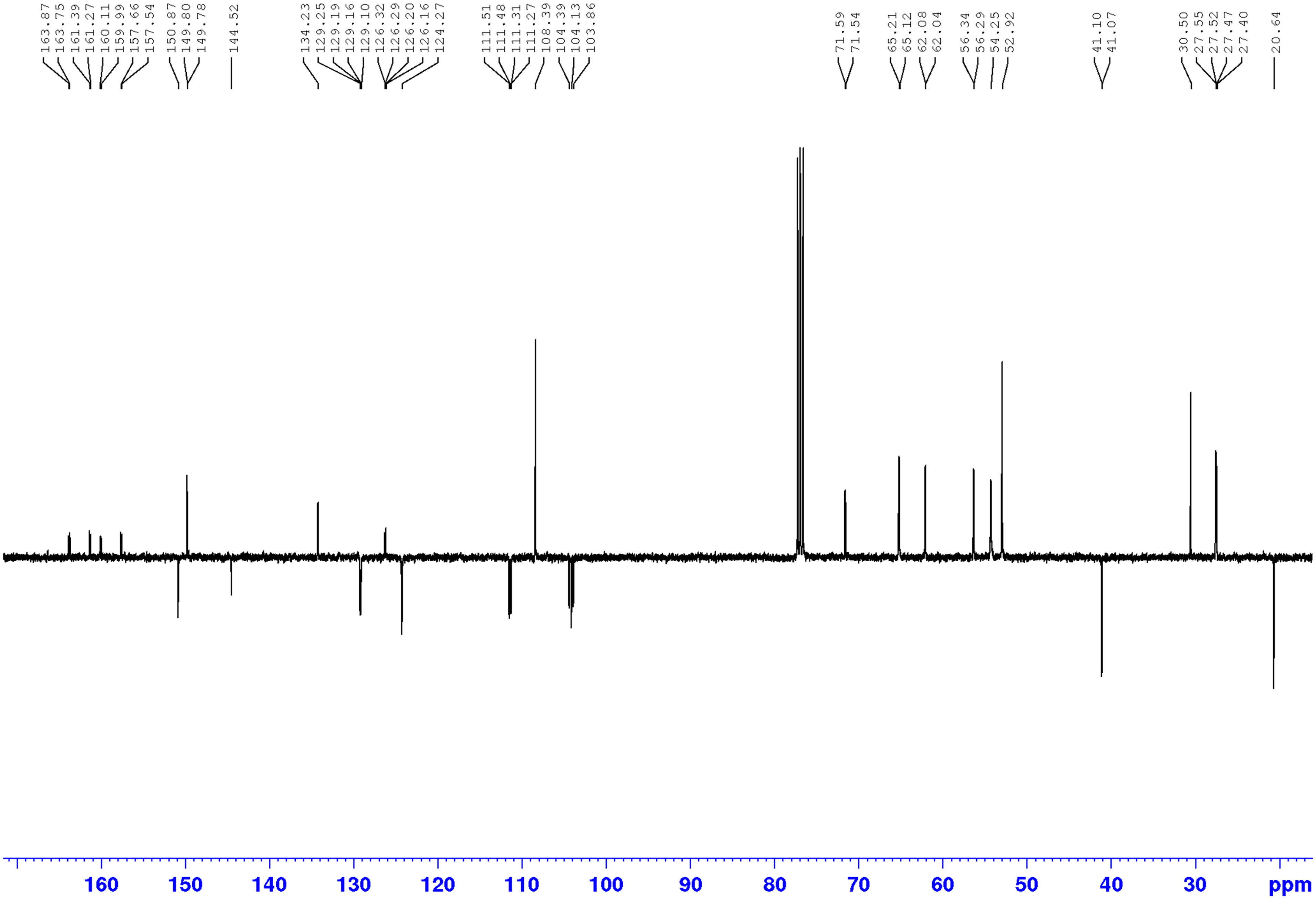

Figure S12. ^{13}C-NMR spectroscopic data for compound **10b**.

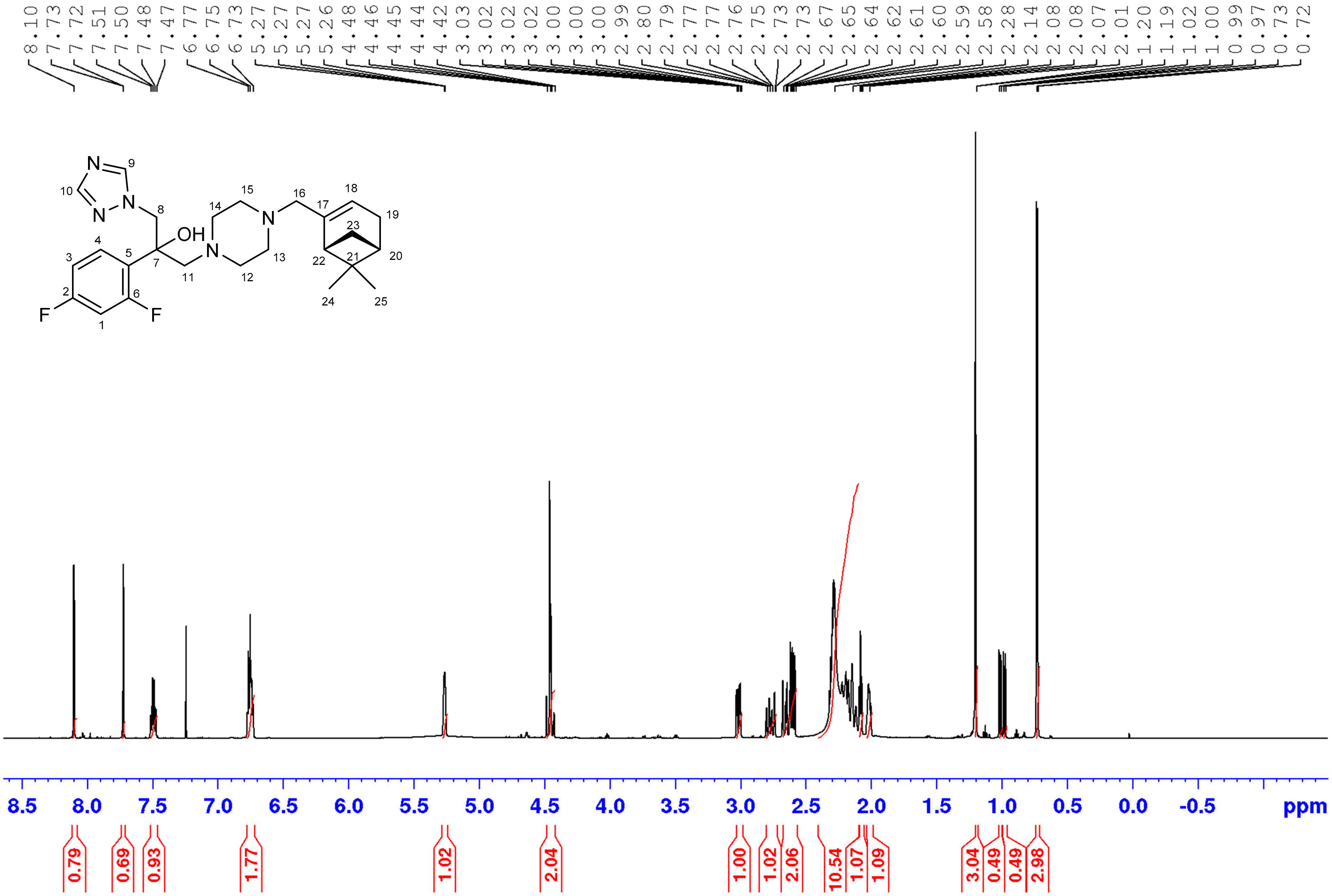

Figure S13. ^{13}C-NMR spectroscopic data for compound **10c**.

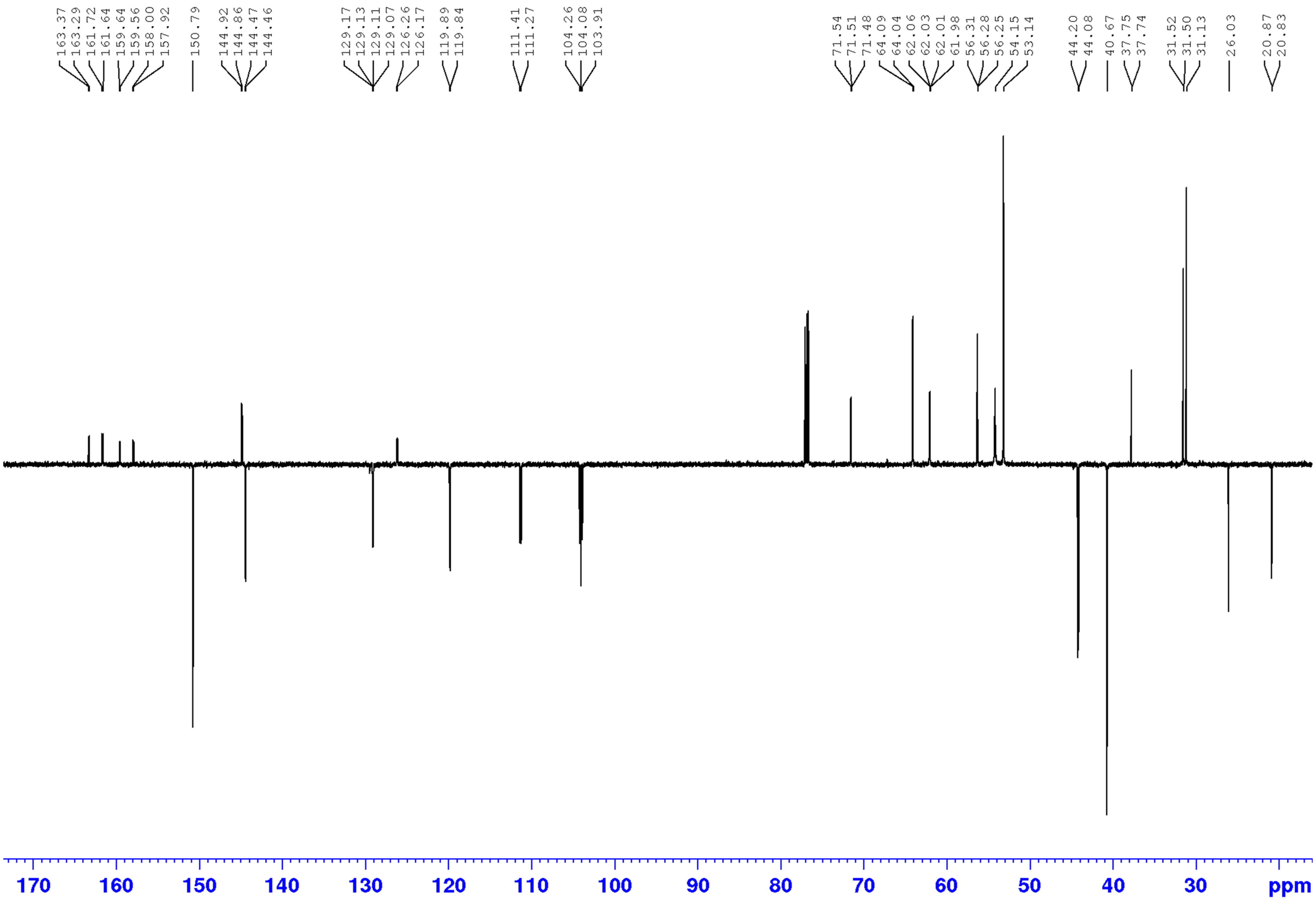

Figure S14. ^{13}C-NMR spectroscopic data for compound **10c**.

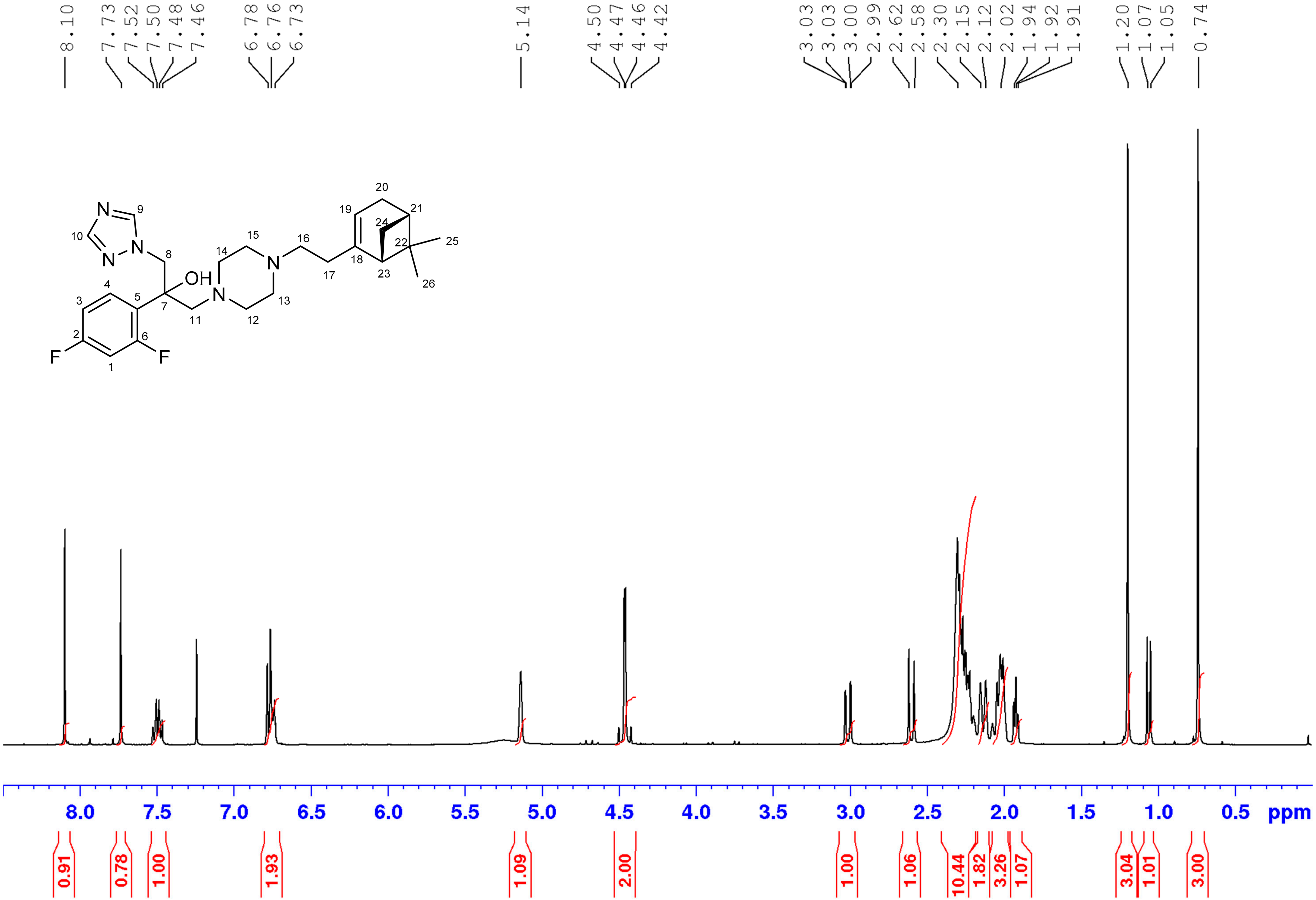

Figure S15. ^{1}H-NMR spectroscopic data for compound **10d**.

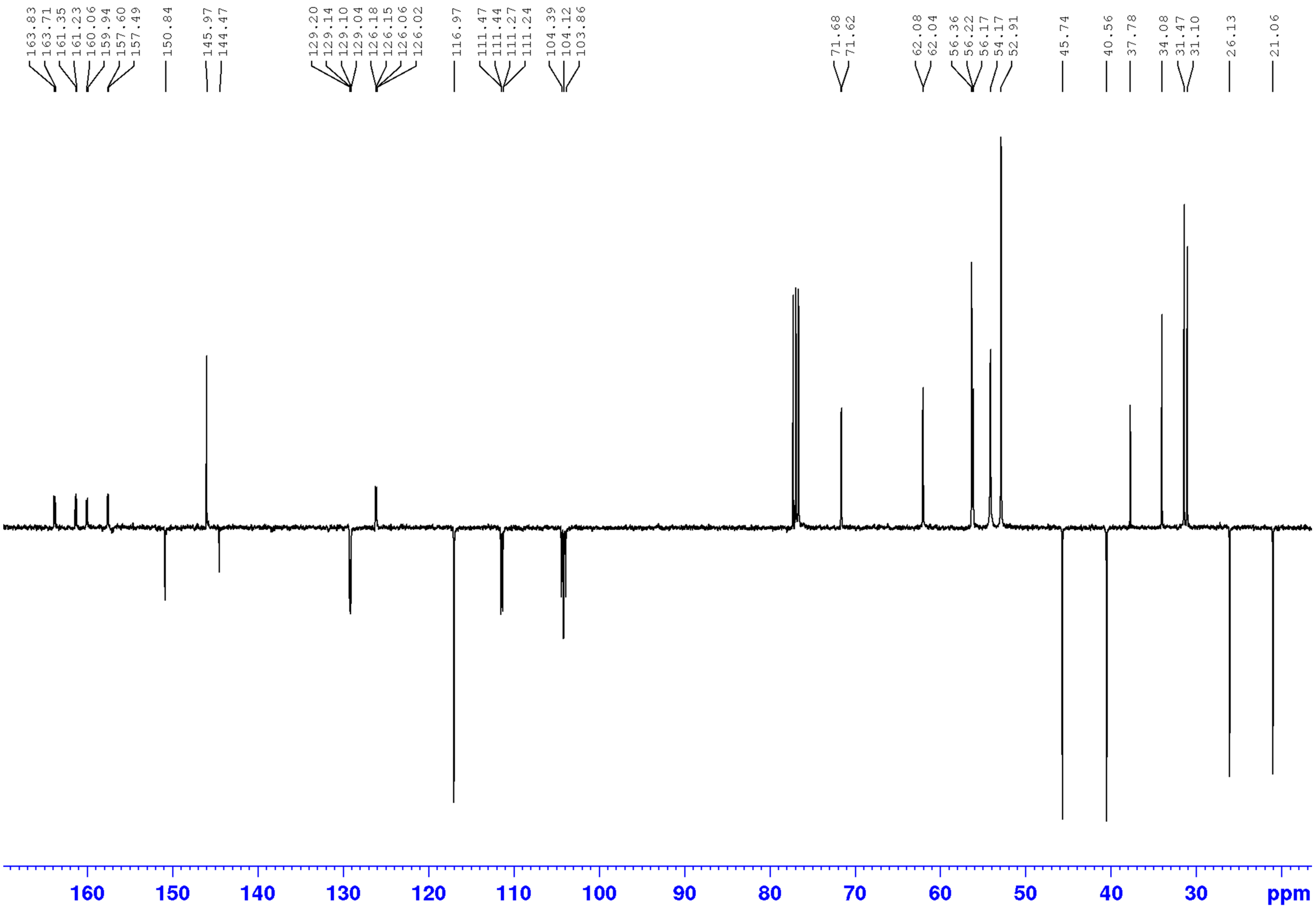

Figure S16. ^{13}C-NMR spectroscopic data for compound **10d**.

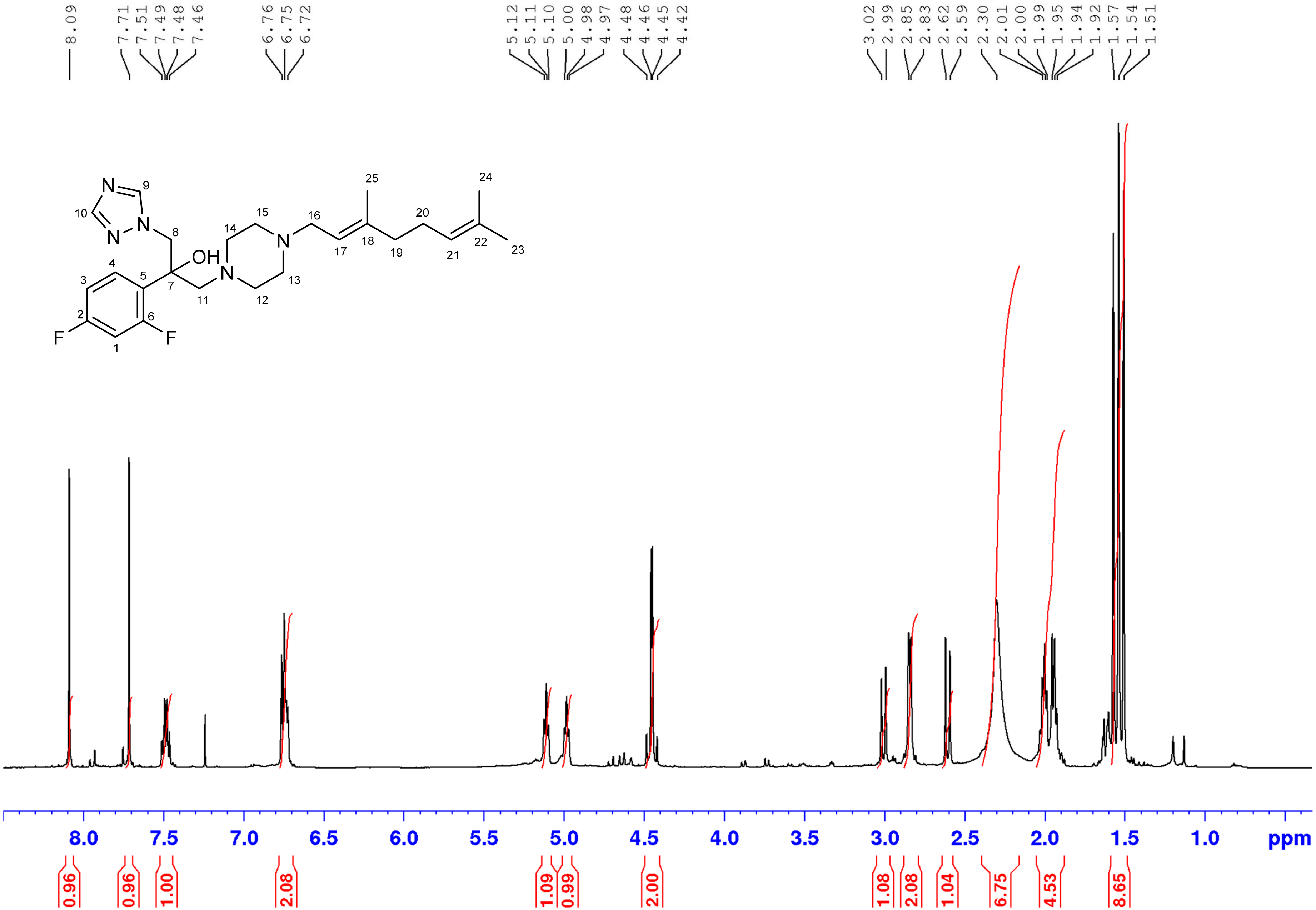

Figure S17. ^{1}H-NMR spectroscopic data for compound **10e**.

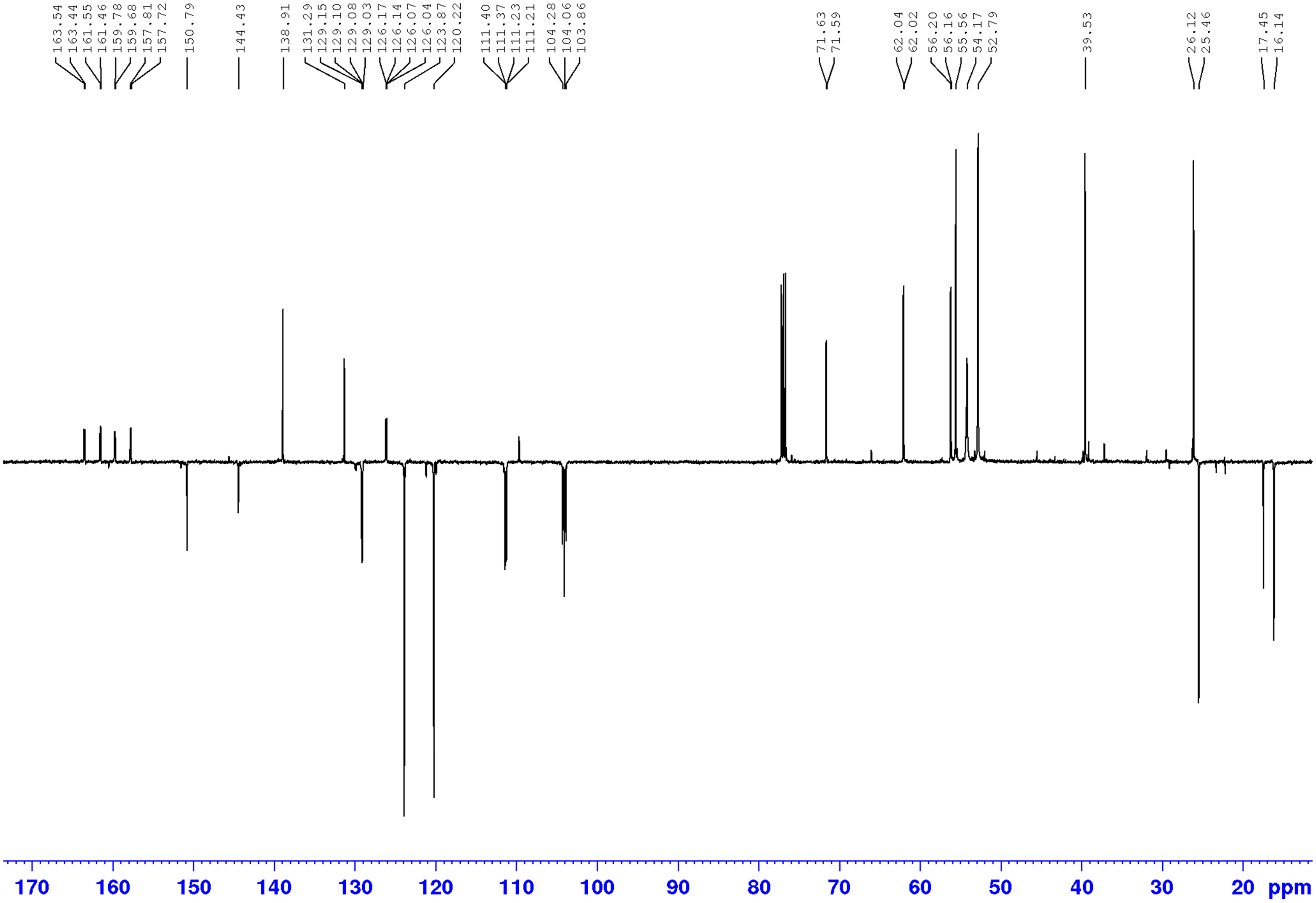

Figure S18. ^{13}C-NMR spectroscopic data for compound **10e**.

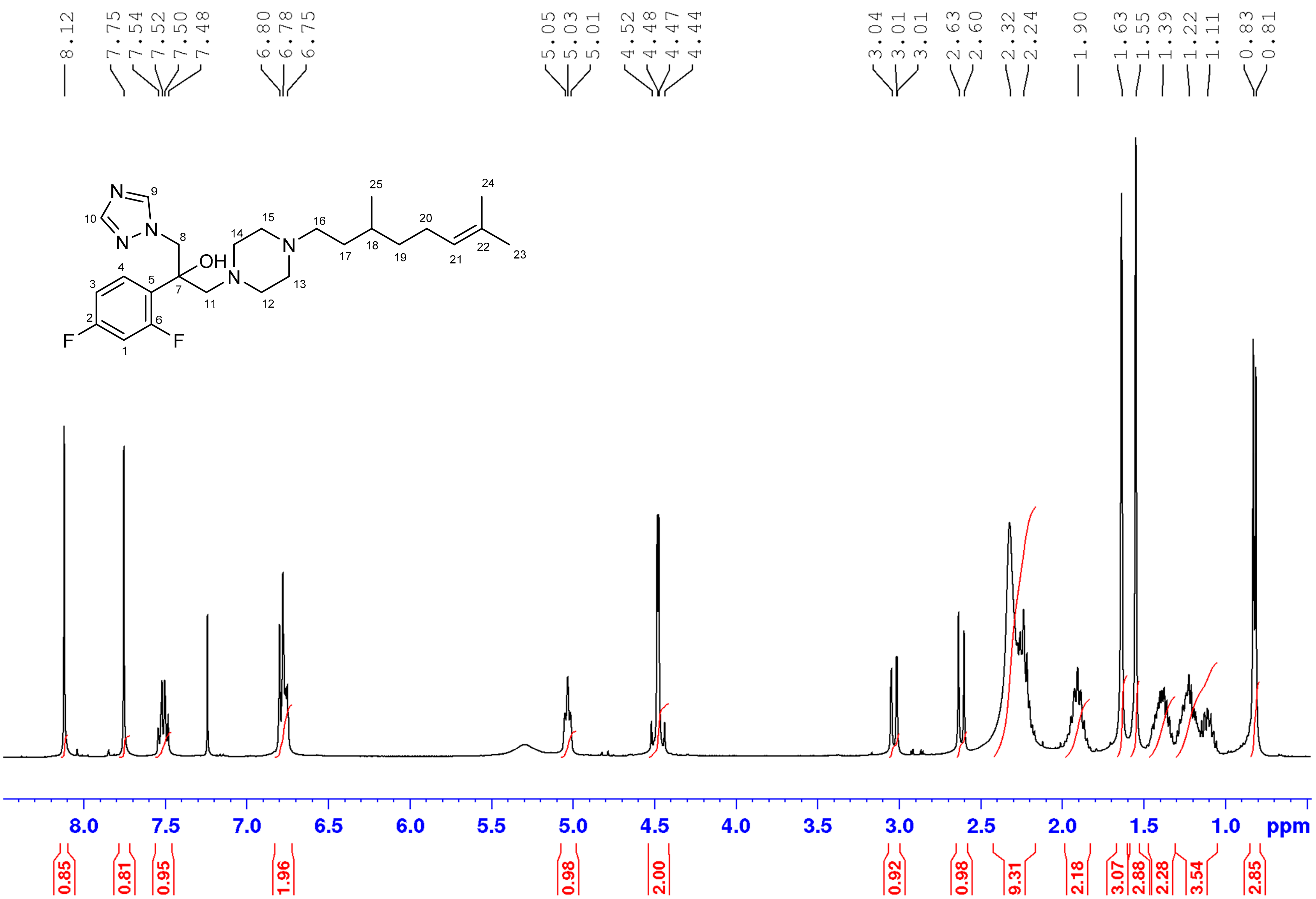

Figure S19. ^{1}H-NMR spectroscopic data for compound **10f**.

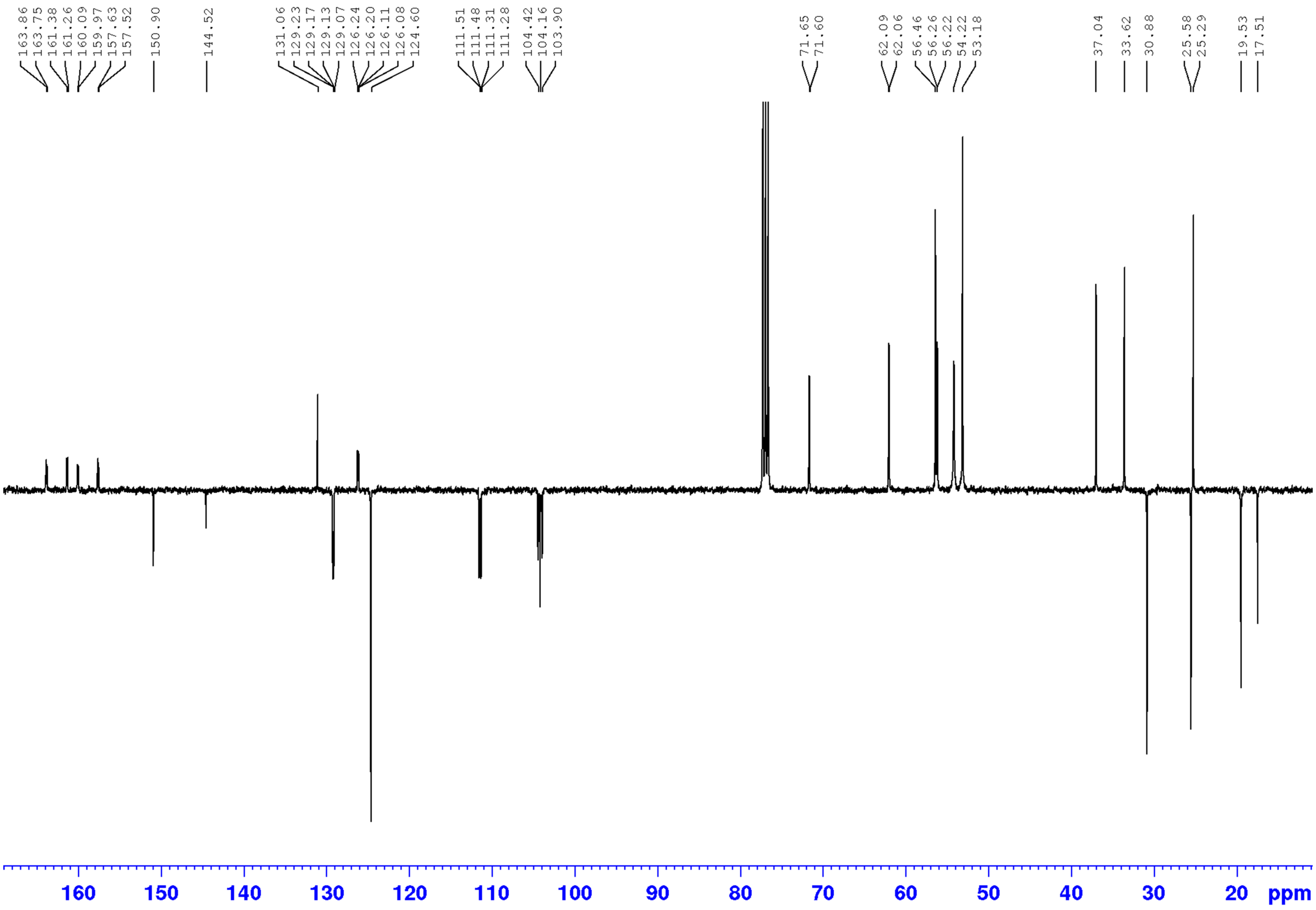

Figure S20. ^{13}C-NMR spectroscopic data for compound **10f**.

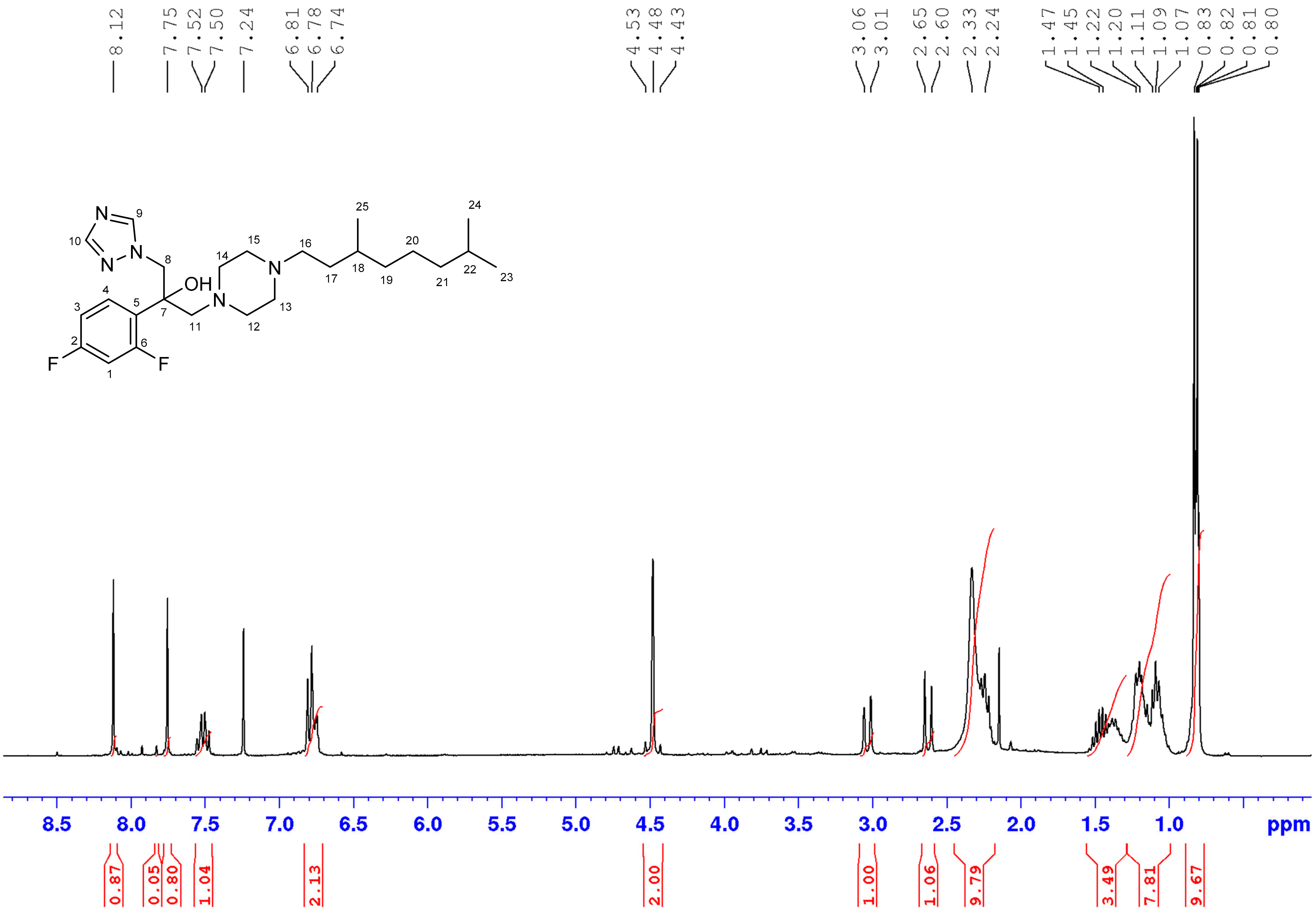

Figure S21. ^{1}H-NMR spectroscopic data for compound **10g**.

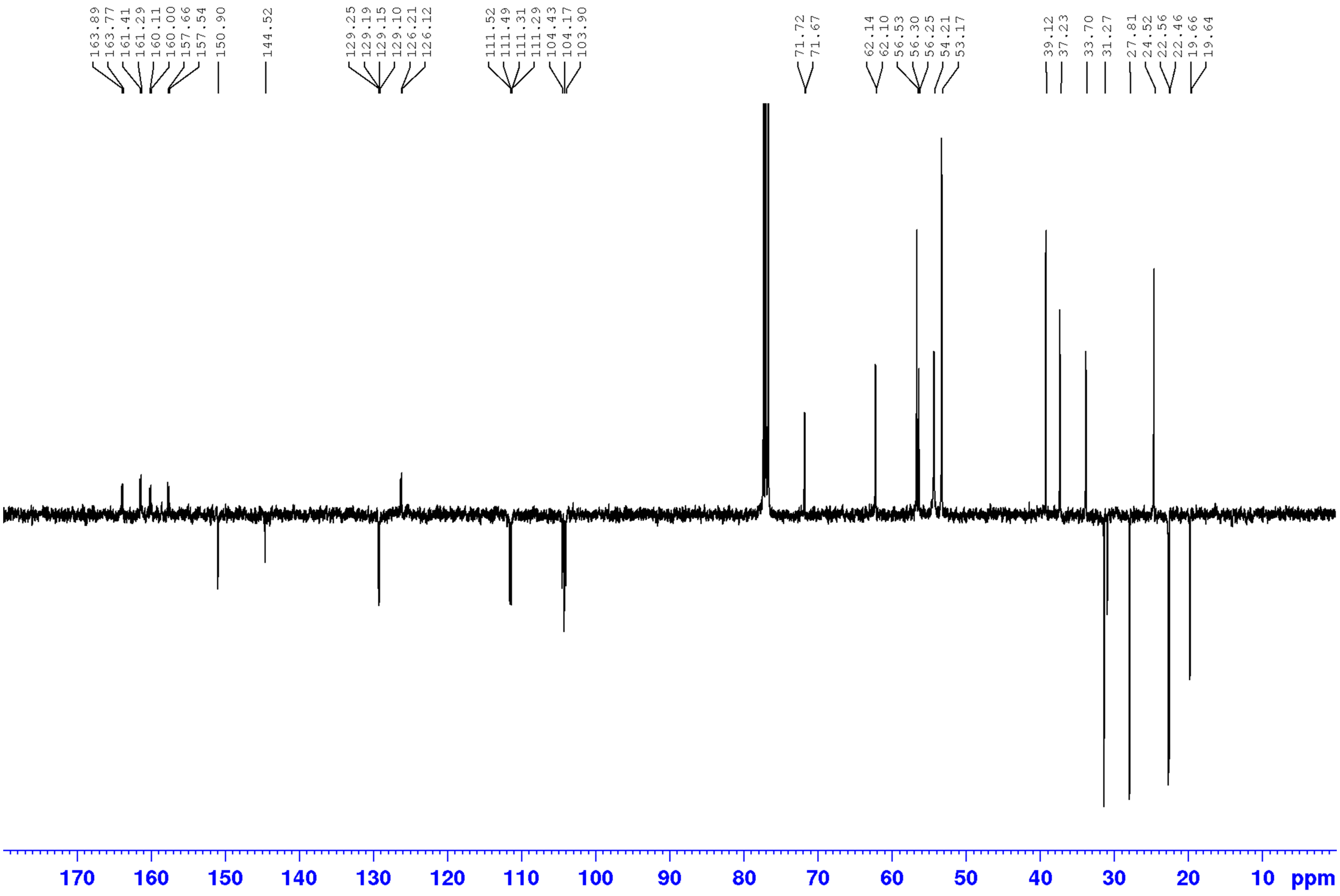

Figure S22. ^{13}C-NMR spectroscopic data for compound **10g**.

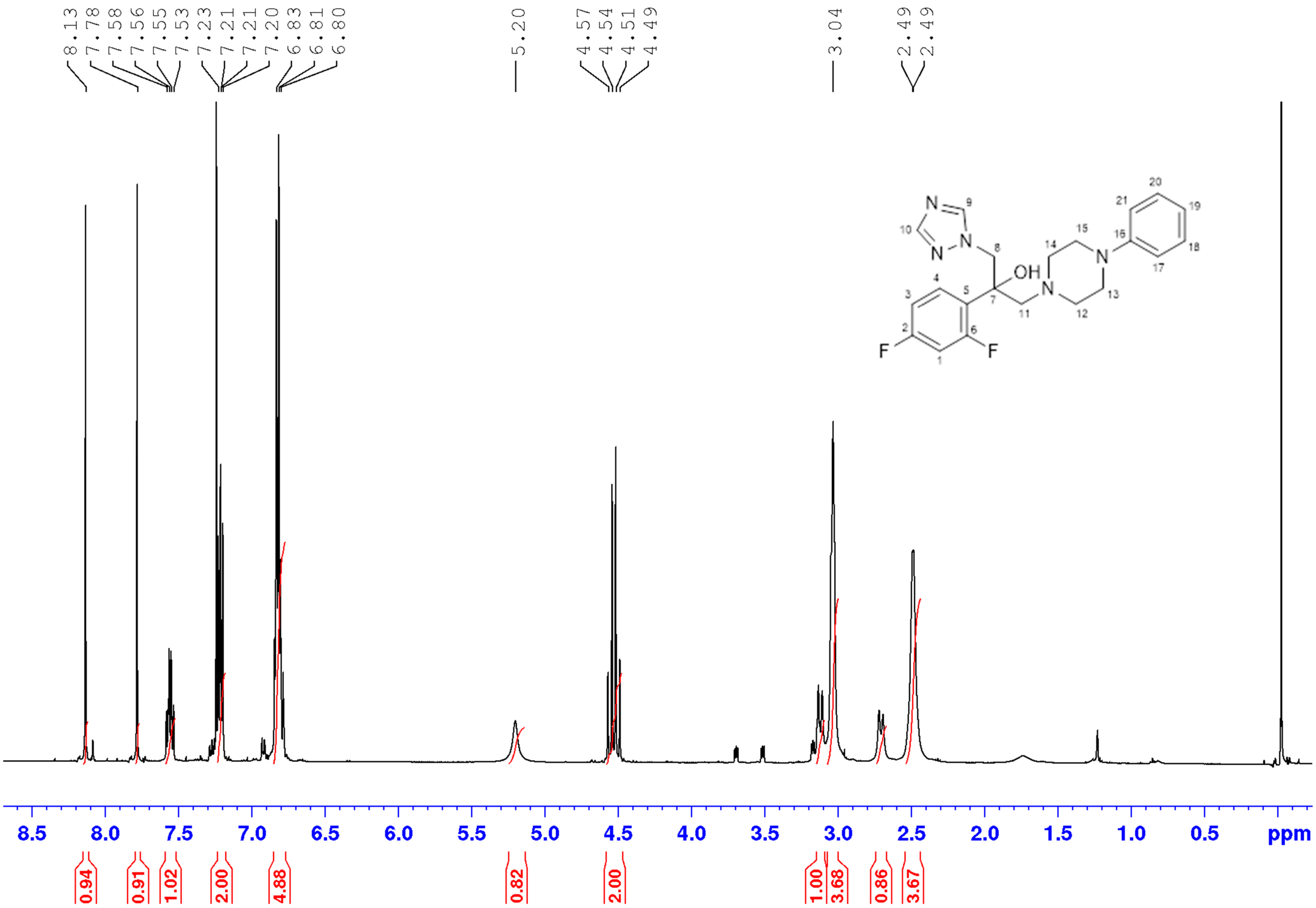

Figure S23. ^{1}H-NMR spectroscopic data for compound **10h**.

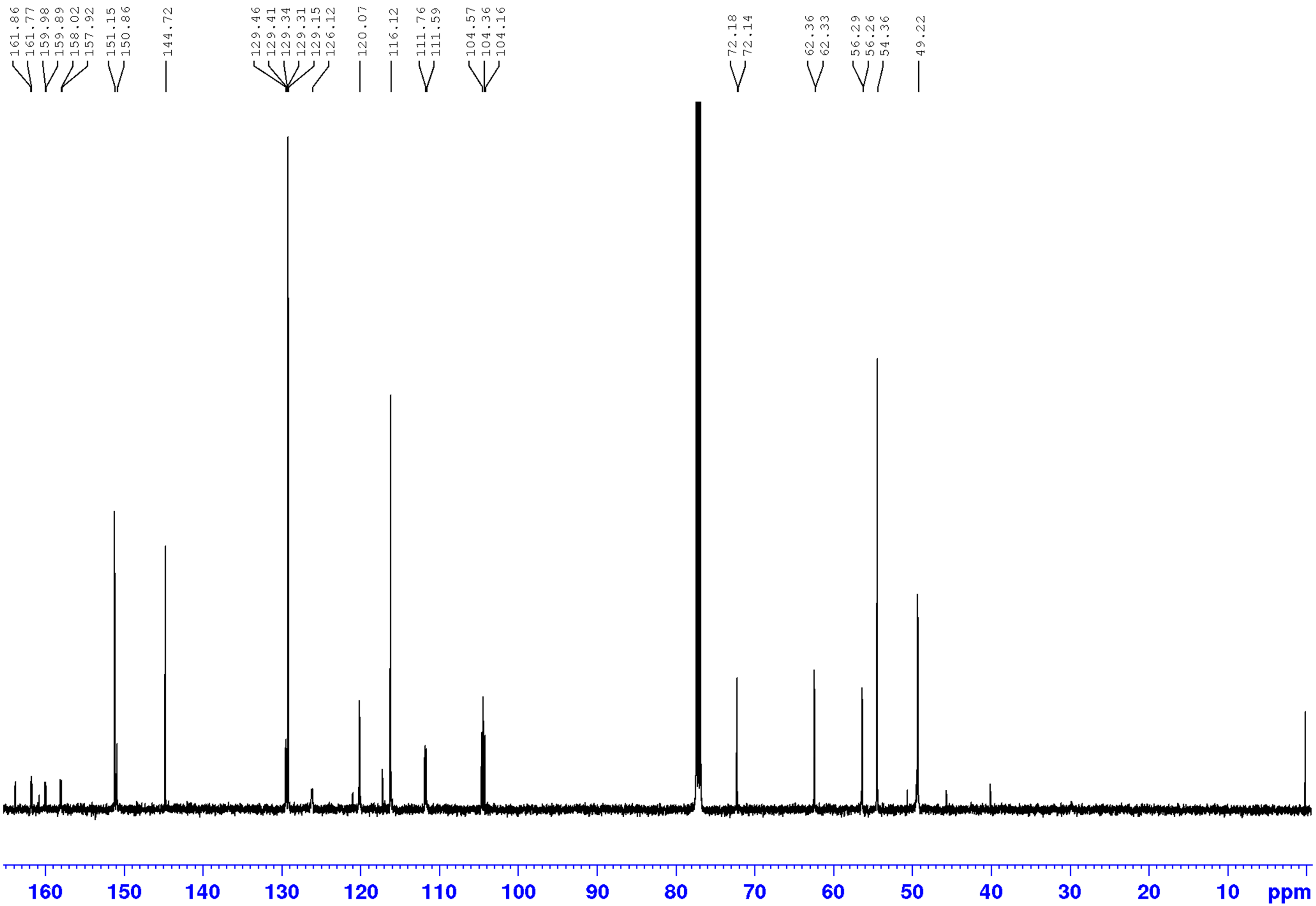

Figure S24. ^{13}C-NMR spectroscopic data for compound **10h**.

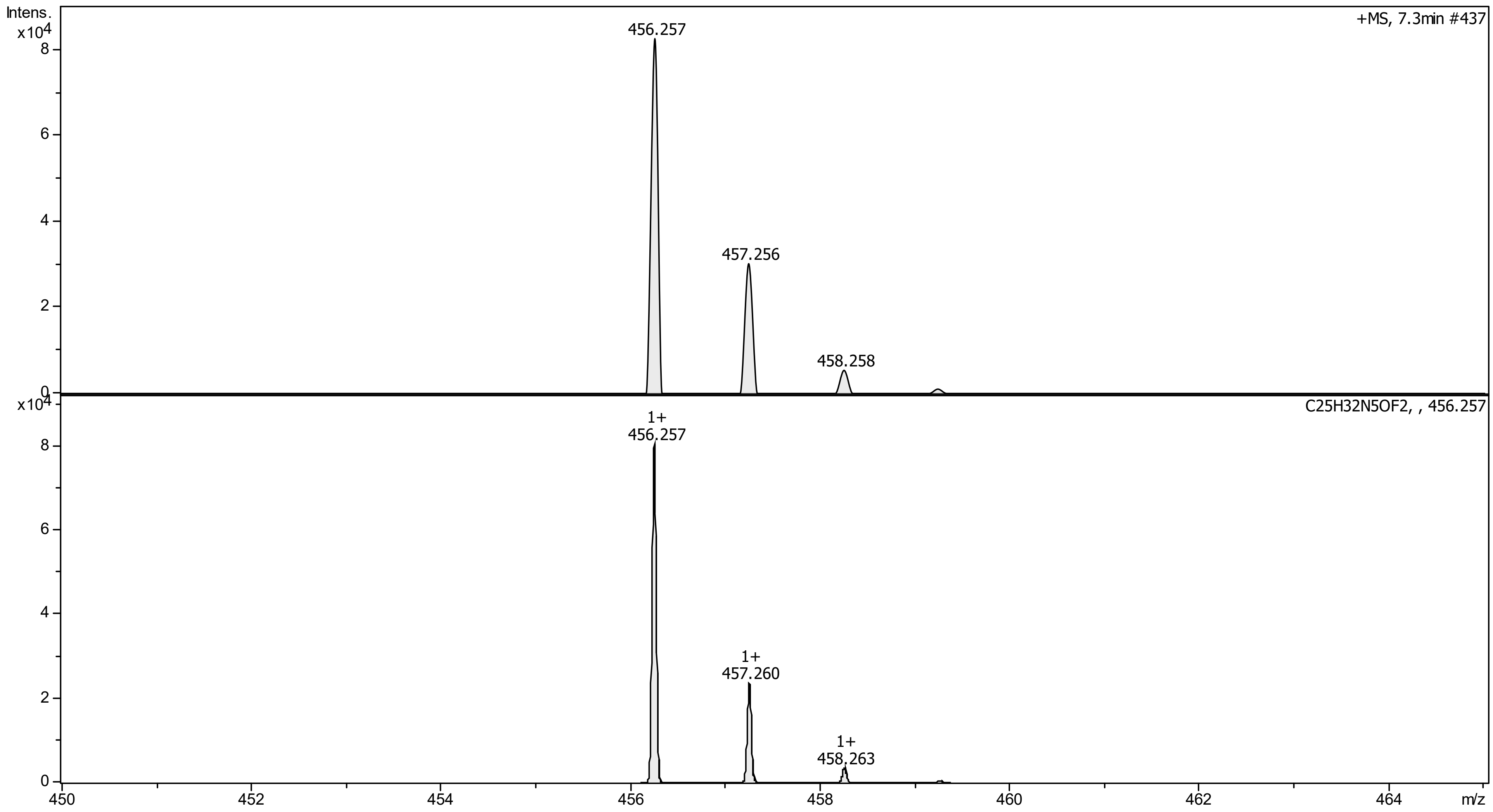

Figure S25. HRMS of the compound **10a** with simulation $C_{25}H_{32}N_5OF_2^+$.

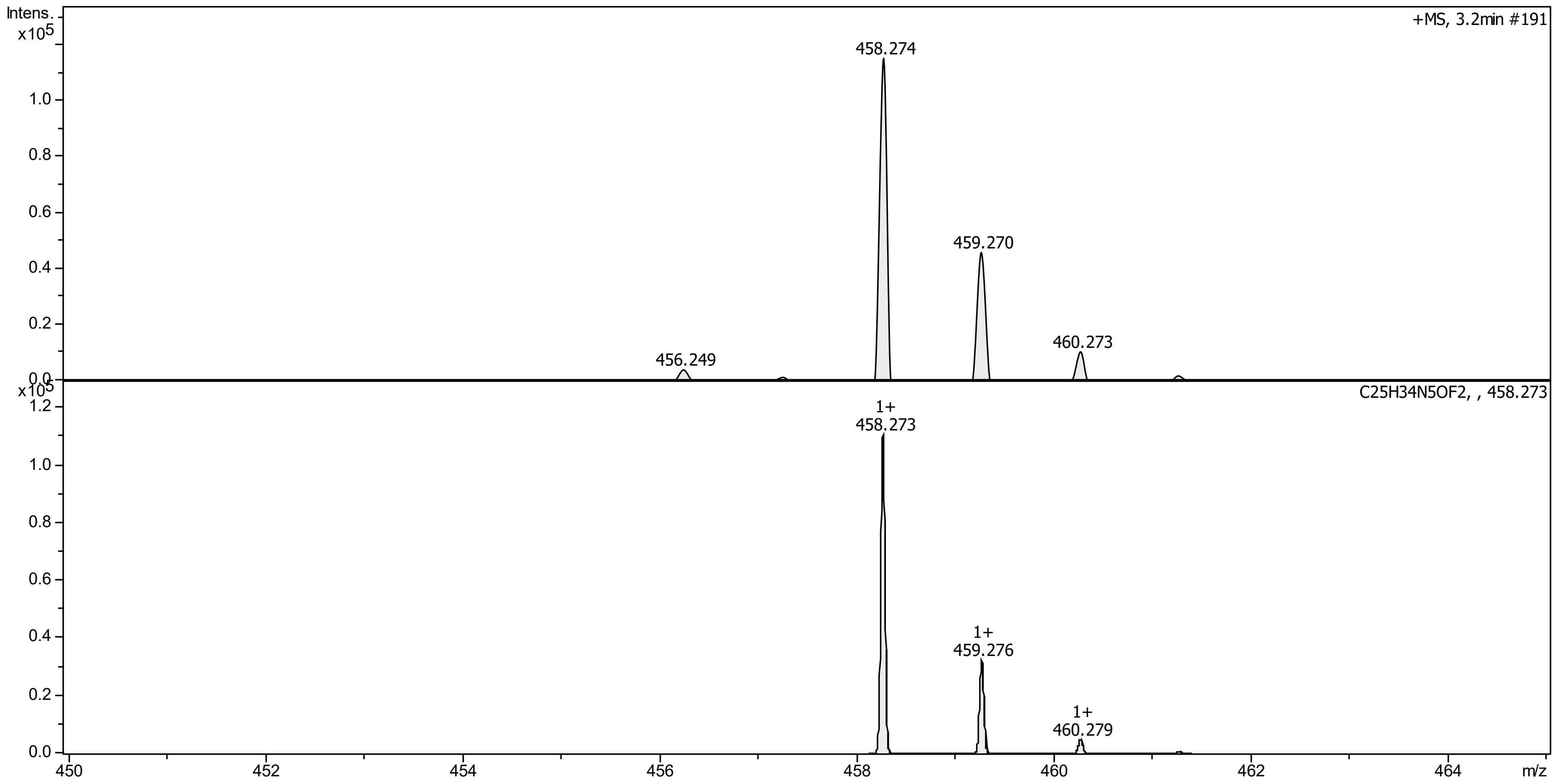

Figure S26. HRMS of the compound **10b** with simulation $C_{25}H_{34}N_5OF_2^+$.

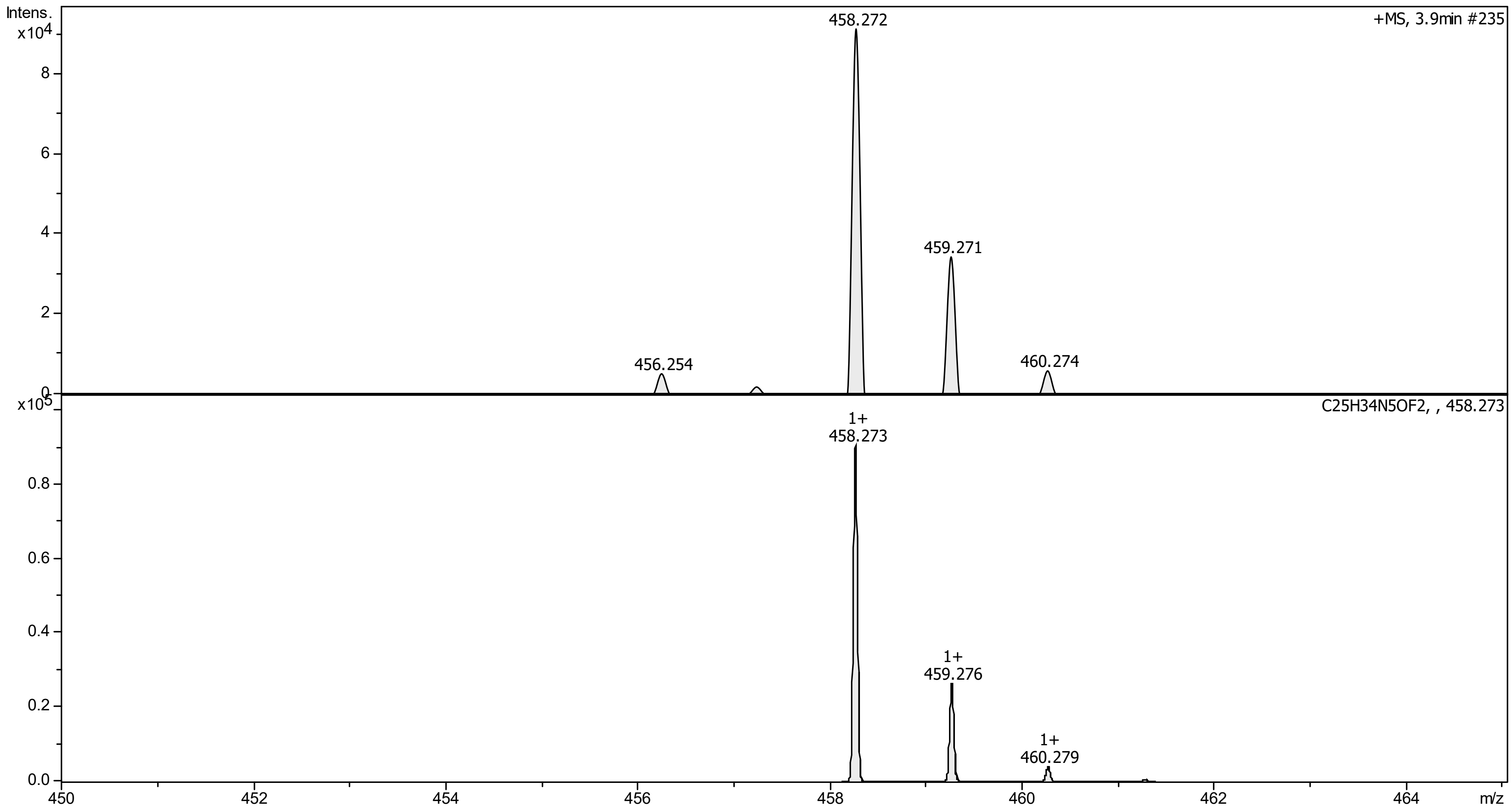

Figure S27. HRMS of the compound **10c** with simulation $C_{25}H_{34}N_5OF_2^+$.

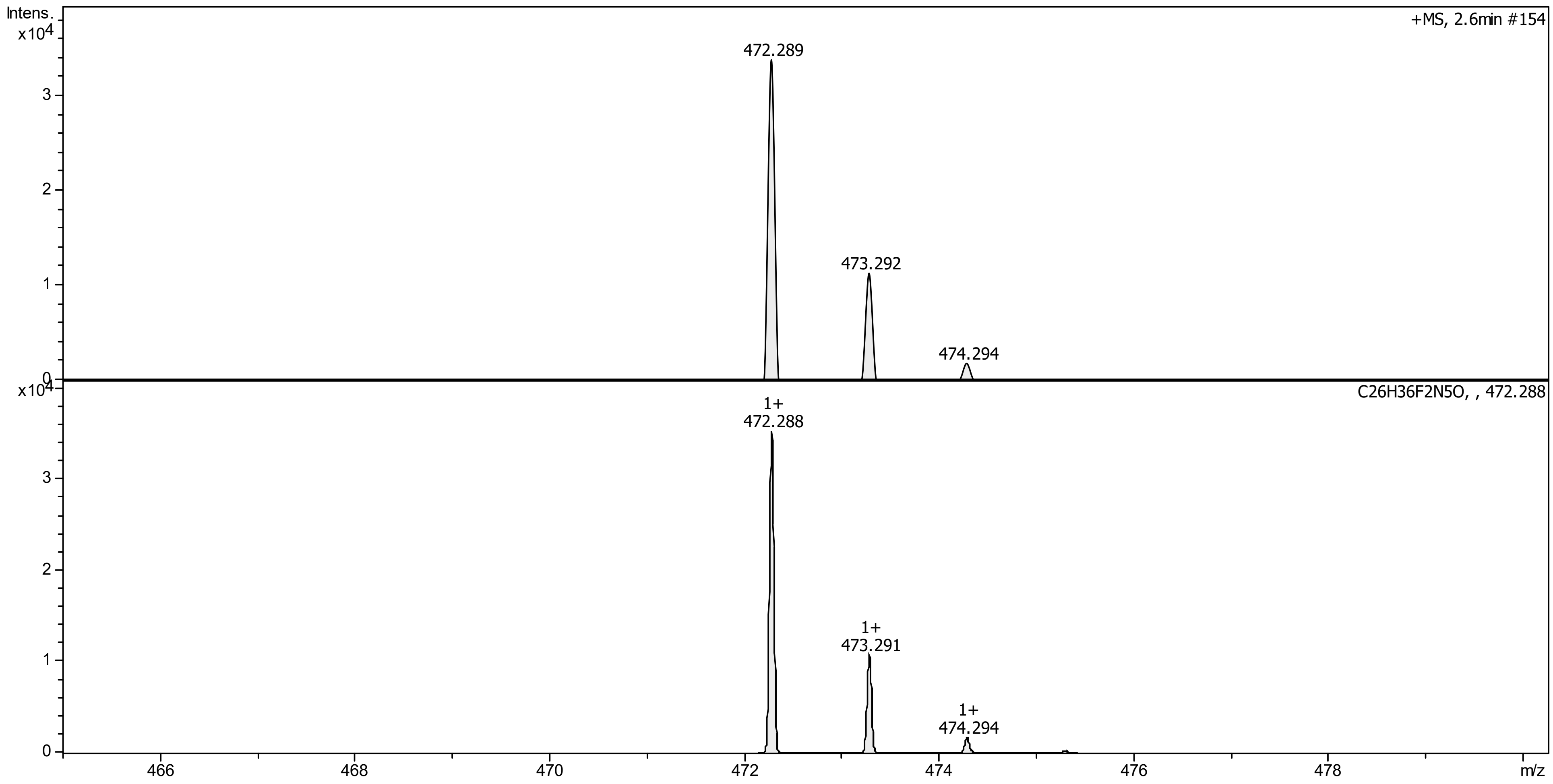

Figure S28. HRMS of the compound **10d** with simulation $C_{26}H_{36}N_5OF_2^+$.

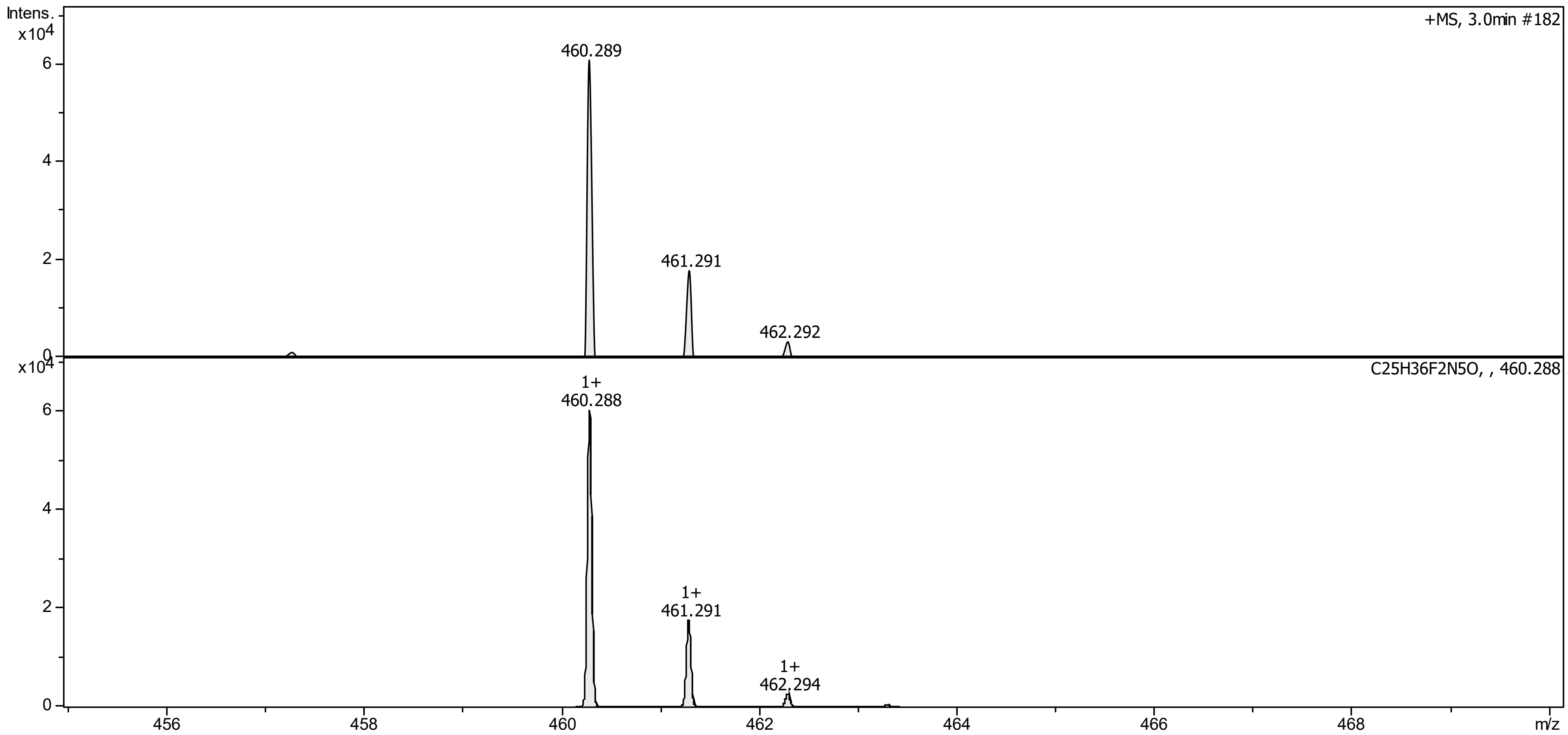

Figure S29. HRMS of the compound **10e** with simulation $C_{25}H_{36}N_5OF_2^+$.

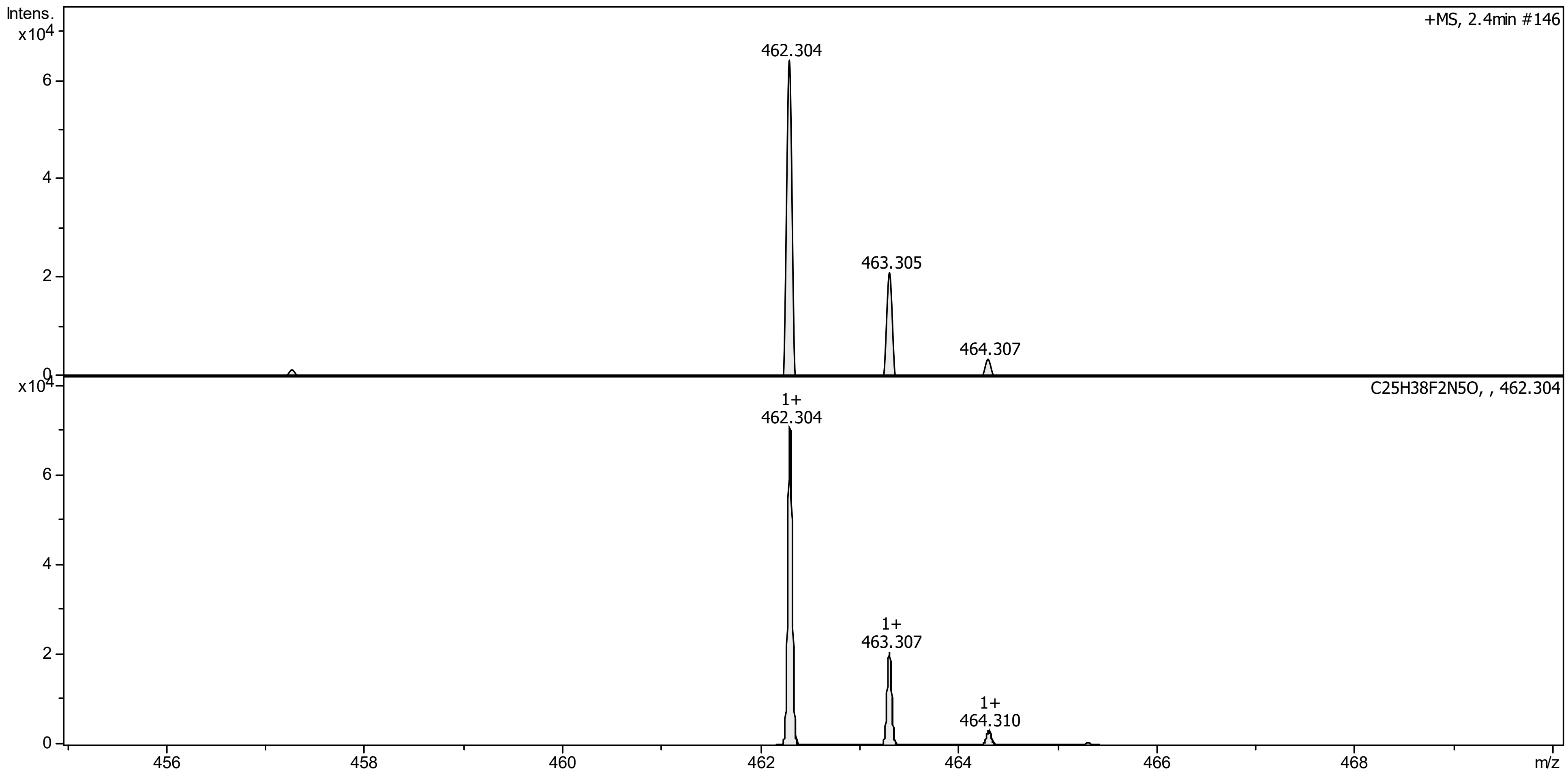

Figure S30. HRMS of the compound **10f** with simulation $C_{25}H_{38}N_5OF_2^+$.

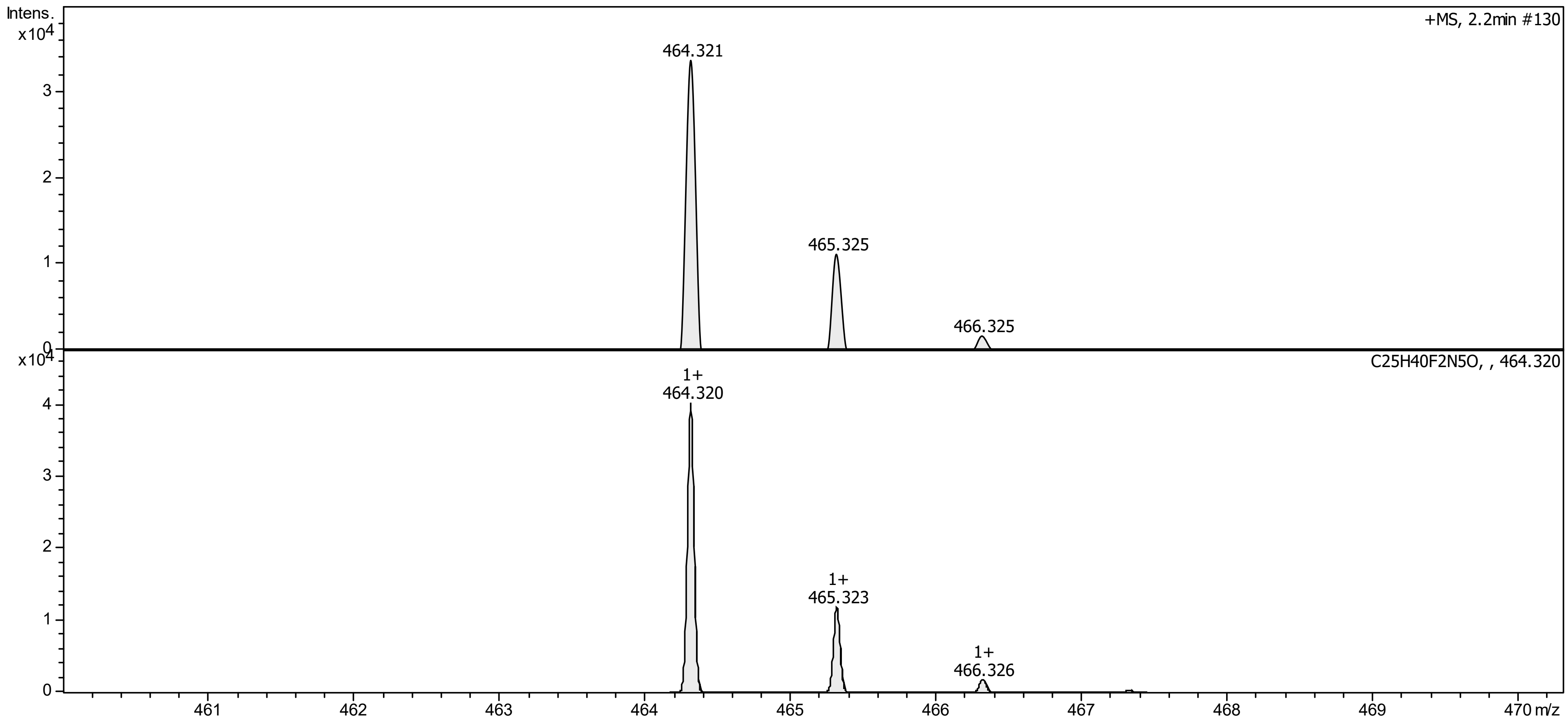

Figure S31. HRMS of the compound **10g** with simulation $C_{25}H_{40}N_5OF_2^+$.

2. Cytotoxicity results

Table S1. Mean standard deviation (S.D.) % viability of murine fibroblasts at 24 and 48 h treated with **10a** and **10h**

	**10a**				**10h**			
Compound	24 h		48 h		24 h		48 h	
conc. (ug/ml)	Mean	S.D.	Mean	S.D.	Mean	S.D.	Mean	S.D.
0.000128	99.03802	5.617851	164.1302	43.49461*	82.776	8.620915*	126.521	25.96578
0.00064	92.4874	4.288911	151.174	36.77111*	83.3257	9.59642*	98.26907	9.298079
0.0032	93.06764	7.575664	130.9426	24.79858*	88.6853	9.287817*	104.8672	10.71842
0.016	88.16613	5.582378*	163.5904	21.23595*	88.28829	7.291397*	109.88	10.43458
0.08	78.80592	5.553963*	143.479	30.51944*	84.30295	4.658406*	100.377	11.61315
0.4	80.60773	10.44168*	164.7558	26.99878*	82.45534	4.530368*	110.9254	6.091775
2	76.83616	7.012305*	166.8038	22.76988*	84.18079	4.41302*	116.2468	14.70779

* p ≤ 0.005

Table S2. Mean ± standard deviation (S.D.) % viability of murine fibroblasts at 24 and 48 h treated with **10d** and **10e**

	**10d**				**10e**			
Compound	24 h		48 h		24 h		48 h	
conc. (ug/ml)	Mean	S.D.	Mean	S.D.	Mean	S.D.	Mean	S.D.
0.015625	94.3118	14.90656	126.4248	16.4335	98.52528	7.367534	94.27441	13.3297
0.03125	96.3764	11.01797	121.438	6.8391	96.8118	11.09797	92.3219	13.5363
0.0625	95.47753	10.41641	110.3562	18.7046	103.736	12.41573	132.2691	18.3723
0.125	98.84831	13.95933	99.68997	21.9126	92.72472	6.418196	99.41293	13.6618
0.25	87.45787	9.808199	103.5752	18.3340	92.72472	8.113975	107.223	8.1963
0.5	82.40169	18.20816	102.0778	11.4795	96.15169	11.07017	113.1332	16.3888
1	93.10393	6.406198	99.62401	19.2648	92.42978	4.663796	109.8681	9.1816
2	76.1236	6.318664*	91.34565	10.6533	94.48034	13.10169	106.9987	5.7246

* p ≤ 0.005

Table S3. Mean ± standard deviation (S.D.) viability % of murine fibroblasts at 24 and 48 h treated with **10f** and **10g**

	**10f**				**10g**			
Compound	24 h		48 h		24 h		48 h	
conc. (ug/ml)	Mean	S.D.	Mean	S.D.	Mean	S.D.	Mean	S.D.
0.015625	110.3933	16.27437	109.69	15.8401	97.72472	10.74494	105.6135	10.3570
0.03125	95.78652	11.90013	111.2665	17.0486	93.69382	3.618366	104.2216	12.6720
0.0625	104.8455	14.14209	114.8483	17.9036	85.35112	3.787971	109.4723	8.7817
0.125	92.16292	8.275098	113.7665	10.3552	85.53371	9.571963	122.9288	19.2565
0.25	100.4775	11.42081	107.5594	13.5086	88.23034	10.46116	101.7942	11.3079
0.5	112.3174	14.28318	114.8417	15.1429	87.82303	5.451537	89.4723	11.3727
1	90.75843	8.251162	119.7493	11.7707	91.74157	5.610294	88.9314	11.8388
2	90.39326	13.65581	102.4604	9.2628	80.23876	12.08256*	70.46834	13.9502*

* p ≤ 0.005

3. Molecular modeling

Table S4. Physicochemical parameters calculated for **10a-h** by SwissADME

Comp.	MW	Fraction Csp3	#Rotatable bonds	#H-bond acceptors	#H-bond donors	MR	TPSA	XLOGP3
**10a**	455.54	0.44	8	7	1	130.96	57.42	3.27
**10b**	457.56	0.52	8	7	1	132.09	57.42	3.22
**10c**	457.56	0.6	7	7	1	130.19	57.42	2.71
**10d**	471.59	0.62	8	7	1	135	57.42	3.17
**10e**	459.58	0.52	10	7	1	134.2	57.42	4.01
**10f**	461.59	0.6	11	7	1	134.68	57.42	4.31
**10g**	463.61	0.68	12	7	1	135.15	57.42	4.76
**10h**	413.46	0.36	7	7	1	116.38	57.42	1.98

Table S5. Solubility, GI apsorption, BBB penetration, Pgp substrate, and CYP inhibitory parameters of **10a-h** calculated by SwissADME

Comp.	ESOL Class	GI absorption	BBB permeant	Pgp substrate	CYP1A2 inhibitor	CYP2C19 inhibitor	CYP2C9 inhibitor	CYP2D6 inhibitor	CYP3A4 inhibitor
**10a**	Moderately soluble	High	Yes	Yes	No	No	No	Yes	Yes
**10b**	Moderately soluble	High	Yes	Yes	No	No	No	Yes	Yes
**10c**	Moderately soluble	High	Yes	Yes	No	No	No	Yes	Yes
**10d**	Moderately soluble	High	Yes	Yes	No	No	No	Yes	Yes
**10e**	Moderately soluble	High	Yes	No	No	No	No	Yes	Yes
**10f**	Moderately soluble	High	Yes	No	No	No	No	Yes	Yes
**10g**	Moderately soluble	High	Yes	No	No	No	No	Yes	Yes
**10h**	Soluble	High	Yes	Yes	No	No	No	Yes	Yes

Table S6. Skin permeation, druglikeness, bioavailability, and PAINS parameters of **10a-h** calculated by SwissADME

Comp.	log Kp (cm/s)	Lipinski #violations	Ghose #violations	Veber #violations	Egan #violations	Muegge #violations	Bioavailability Score	PAINS #alerts	Brenk #alerts
**10a**	-6.76	0	1	0	0	0	0.55	0	0
**10b**	-6.8	0	1	0	0	0	0.55	0	1
**10c**	-7.17	0	1	0	0	0	0.55	0	1
**10d**	-6.93	0	1	0	0	0	0.55	0	1
**10e**	-6.26	0	1	0	0	0	0.55	0	1
**10f**	-6.06	0	1	1	0	0	0.55	0	1
**10g**	-5.75	0	2	1	0	0	0.55	0	0
**10h**	-7.42	0	0	0	0	0	0.55	0	0
